# The effects of health facility access and quality on family planning decisions in urban Senegal

**DOI:** 10.1002/hec.3615

**Published:** 2017-11-02

**Authors:** Christopher J. Cronin, David K. Guilkey, Ilene S. Speizer

**Affiliations:** ^1^ Department of Economics University of Notre Dame Notre Dame IN USA; ^2^ Department of Economics and Carolina Population Center University of North Carolina at Chapel Hill Chapel Hill NC USA; ^3^ Gillings School of Public Health, Department of Maternal and Child Health and Carolina Population Center University of North Carolina at Chapel Hill Chapel Hill NC USA

**Keywords:** discrete factor random effects, endogenous program placement, family planning decisions, health facility quality, urban health programs

## Abstract

Research in developing countries is rarely focused on examining how supply side factors affect family planning decisions due to a lack of facility‐level data. When these data exist, analyses tend to focus on rural environments. In this paper, we study the effects that health facility access and quality have on contraceptive use and desired number of children for women in urban Senegal. Unlike related studies focusing on rural environments, we find no evidence that greater access to health facilities and pharmacies increases contraceptive use among urban women. However, we do find that contraceptive use among urban women is higher with greater facility quality. For example, we find that increasing the proportion of pharmacies employing multiple pharmacists from 0% to 50% would increase contraceptive use by 6.0 percentage points, and increasing the proportion of facilities with family planning guidelines/protocols from 50% to 100% would increase use by 2.1 percentage points.

## 1 INTRODUCTION

Family planning (FP) leads to significant health benefits for women and children (Cleland, Conde‐Agudelo, Peterson, Ross, & Tsui, 2012; Ezeh, Bongaarts, & Mberu, 2012; Tsui, McDonald‐Mosley, & Burke, 2010) and economic and educational benefits for families and communities (Canning & Schultz, 2012). However, sub‐Saharan Africa and the West African region in particular lag in the use of FP (Cleland, Ndugwa, & Zulu, 2011; Khan, Mishra, Arnold, & Abderrahim, 2007). Although there is an abundance of research on FP decisions in sub‐Saharan Africa (Cleland et al., 2011; Wang, Wang, Pullum, & Ametepi, 2012), the effects of supply‐side factors are not often studied due to lack of facility‐level data 1The small number of studies that use facility‐level data most often use DHS Service Provision Assessment data linked with individual‐level DHS data. See, for example, Feyiestan and Ainsworth (1996), Chen and Guilkey (2003), Cohen (2000), and Arends‐Kuenning and Kessy (2007)., and if data are available, analyses frequently focus on rural environments (Angeles, Guilkey, & Mroz, 2005; Arends‐Kuenning & Kessy, 2007; Chen & Guilkey, 2003; Feyisetan & Ainsworth, 1996) and only a small number examine the determinants of FP use in urban settings (Ezeh, Kodzi, & Emina, 2010; Khan et al., 2007; Onwuzurike & Uzochukwu, 2001; Zulu, Nii‐Amoo Dodoo, & Ezeh, 2002). The focus on rural environments is due in part to the fact that measurement of supply‐side factors is less challenging in rural areas than in densely populated urban areas.

In this research, we study the effects of health facility access and quality on contraceptive use and desired number of children in urban Senegal. The study of access and quality is particularly important in urban areas of Senegal, where the population is growing rapidly and women (and couples) are more likely to have lower fertility desires and greater need for FP (ANSD, ICF International, 2016). Senegal's population nearly doubled from 1988 to 2010 when it increased from 6.9 million to an estimated 13 million people. The total fertility rate nationally is 5.0; in urban areas, it is 3.9 (ANSD, ICF International, 2012). FP usage is relatively low, as 21.2% of women in union ages 15–49 were using a modern method in 2015, whereas another 21–23% of women in union reported a desire to space or stop childbearing but were not using any method of FP (ANSD, ICF International, 2016). The region of Dakar represents 0.3% of the surface area of the country but about 23% of the total population and 75% of the urban population (ANSD, ICF International, 2012). It also has the highest concentration of public and private health care facilities; despite this, less than 18% of Dakar's female population 15–49 (31% of those in a union) currently uses contraceptives.

We address several empirical and methodological challenges in studying the relationship between contraceptive supply and demand. First, women living in urban environments may seek services from any number of local health facilities; therefore, it is not clear how the supply of FP services should be measured. Studies of urban/rural differentials typically point to access to services as the most important determinant of urban/rural differences (see, for example, Cleland et al., 2006). In rural environments, there is often only one health facility within a 5‐ or 10‐km radius of a woman's home, which simplifies both the collection of facility‐level data and measurement of FP services (Hong, Montana, & Mishra, 2006). Urban environments contain many facilities from which women can obtain services. Therefore, the measurement of the supply side environment requires data from a much larger number of facilities. Moreover, how one should measure access to and quality of these facilities is less obvious. To address this issue, we utilize survey data that includes a complete census public health facilities (hospitals, health centers, and clinics) as well as private facilities (hospitals and clinics) and pharmacies offering reproductive health services in our study area. 2In general, public facilities offer more family planning methods including pills, condoms, injectable contraception, implants, and IUDs. Private facilities generally offer pills, condoms, and injectables but are less likely to offer implants and IUDs. Pharmacies generally offer pills, condoms (male only), injectables, and emergency contraception (MLE, ISSU, 2012a). Under the assumption that distance is an important factor in determining where women seek health services, an assumption that is supported by related work in urban Senegal (Cronin, Guilkey, & Speizer, 2017), we examine empirically the best way to define a woman's service environment by testing several distance bounds around her home. Access is then measured by the number of facilities and services offered, within these bounds, whereas quality is measured by average facility characteristics.

Though related papers often focus exclusively on contraceptive use, we also study the impact that supply‐side factors have on the “ideal” number of children reported by women. In addition to being an interesting policy variable (Castle, 2003; Cohen, 2000), we include it to try to disentangle the channels through which supply‐side factors influence contraceptive use. Our model allows supply‐side factors to impact the ideal number of children and contraceptive use, while controlling for the effect that the ideal number of children has on contraceptive use. This introduces two methodological challenges. First, a woman's fertility desires are endogenous to her decision to use FP. Second, previous research on the association of ideal family size and contraceptive use has struggled with the coding and analysis of ideal family size in contexts such as Senegal with large numbers of non‐numeric responses—answers such as “up to God” or “don't know” are common. In some cases, the non‐numeric option is dropped from the analysis, which leads to selection bias as women who give non‐numeric responses are different than women who give numeric responses (Olaleye David, 1993; Saila‐Ngita, Bravo‐Ureta, & Pérez‐Escamilla, 2003). Another strategy is to code non‐numeric responses at the highest ideal family size category (Riley, Hermalin, & Rosero‐Bixby, 1993). This can bias the results by over‐emphasizing women who want many children. A third approach is to recode non‐numeric responses to the sample mean (Upadhyay & Karasek, 2012). This approach assumes that the unobserved characteristics of non‐numeric responders are similar to those of numeric responders, which is unlikely (Olaleye David, 1993; Saila‐Ngita et al., 2003). The fourth approach, which is adopted in this research, is to keep non‐numeric responders in the analysis as a dummy category so that they can be compared to the responders in the other numeric categories. This imposes fewer assumptions than alternative methods but requires modeling the up to God response jointly with the numerical ideal number of children response. We address these issues by jointly modelling the numerical ideal number of children response and selection into a numerical response.

Our results show that access by itself does not impact FP decisions for these urban women; however, several measures of health facility quality are important factors. The paper concludes with a series of specification tests that both support our empirical strategy and provide insight for future empirical research on fertility and FP in developing countries.

## 2 METHODS

We jointly estimate a set of three equations of the following form:

(1) $$\ln\left[\frac{P\left(G_{\mathrm{ij}}=1\right)}{P\left(G_{\mathrm{ij}}=0\right)}\right]=X_{\mathrm{ij}}^{G}\beta_{G}+H_{j}^{G}\lambda_{G}+\alpha_{j}^{G}+\epsilon_{\mathrm{ij}}^{G},$$

where the dependent variable is the log odds that woman *i* (*i* = 1, 2, …, *N*) from community *j* (*j* = 1, 2, …, *M*) responded that she would “leave it up to God” (about 20% of the sample) when asked her ideal family size. The *X* represents individual‐level variables such as age and education that may affect the outcome. The *H* describes health facilities and pharmacies within an empirically determined distance bound of where the respondent lives. The *α* and *ϵ* represent unobserved heterogeneity at the community and individual levels, respectively.

The second equation models self‐reported ideal number of children for women that provide a numeric response:

(2) $$C_{\mathrm{ij}}=X_{\mathrm{ij}}^{C}\beta_{C}+H_{j}^{C}\lambda_{C}+\alpha_{j}^{C}+\epsilon_{\mathrm{ij}}^{C}.$$

Independent variables in this equation are identical to those in Equation (1). The final equation models contraceptive use

(3) $$\ln\left[\frac{P\left(F_{\mathrm{ij}}=1\right)}{P\left(F_{\mathrm{ij}}=0\right)}\right]=C_{\mathrm{ij}}^{F}\gamma_{F}+G_{\mathrm{ij}}^{F}\delta_{F}+X_{\mathrm{ij}}^{F}\beta_{F}+H_{j}^{F}\lambda_{F}+\alpha_{j}^{F}+\epsilon_{\mathrm{ij}}^{F},$$

where the dependent variable is the log odds that woman *i* from community *j* uses any FP method. Note that a woman's ideal number of children or up to God response is allowed to affect this outcome through *C* and *G*, respectively.

Estimation is complicated by several factors. First, several studies provide strong evidence that health programs and facilities are often targeted to high need communities (e.g., Angeles, Guilkey, & Mroz, 1998; Gertler & Molyneaux, 1994; Pitt, Rosenzweig, & Gibbons, 1993; Rosenzweig & Wolpin, 1986). The statistical implication of this fact is that health facility variables, *H*, may be correlated with unobservable fixed characteristics of the communities, *α*. As a result, we include community‐level dummy variables representing the 41 communities from which individuals are selected. These community‐level fixed effects provide a nonparametric solution to the program‐targeting problem.

To control for the endogeneity of ideal family size and the up to God response, we allow the *ϵ*'s in the equations to be correlated and estimate the system of equations by full information, maximum likelihood. All parameters are technically identified by the nonlinearity of the model; however, we exclude whether the household employs outside help, has indoor plumbing for drinking water, has indoor toilet plumbing, the number of bedrooms in the woman's home, and the number of health facilities within a distance bound that offer delivery services from the FP equation. In Section 3.5, we test the validity of these exclusion restrictions.

We do not make specific distributional assumptions about *ϵ* but rather use a Heckman and Singer (1984) type discrete factor approach where the joint distribution of (*ϵ*^*G*^, *ϵ*^*C*^, *ϵ*^*F*^) is estimated along with the other parameters of the model. Each error component, *ϵ*, is constructed as

(4) $$\epsilon_{\mathrm{ij}}=\mu_{i}+\eta_{\mathrm{ij}},$$

where *μ* captures common unobserved heterogeneity across the three equations and *η* is the remaining independent, identically distributed (i.i.d) error term. The distribution of *μ* is approximated by a discrete function such that, after selecting the number of points of support (discussed in Section 3.4), the location and probability of support points is estimated. We assume that *η* follows a logistic cumulative distribution in the up to God and FP equations and a normal distribution in the ideal number of children equation.

This method allows for very general patterns of error correlations across equations and it also tends to do better than parametric maximum likelihood when there are weak instruments (Guilkey & Lance, 2013; Mroz, 1999). Another possible method of estimation that does not require normality assumptions is instrumental variables. Unfortunately, standard instrumental variables methods would not control for the sample selectivity problem that we face here.

## 3 DATA SET AND RESULTS

### 3.1 Survey details

Data come from baseline household and facility data collected in 2011 by the Measurement, Learning & Evaluation (MLE) Project in Senegal. Data were collected from six urban sites: Dakar, Guédiawaye, Pikine, Mbao, Mbour, and Kaolack. A multistage sampling design was used to select a representative sample of women ages 15–49. In total, 9,614 women (88.9% response rate) responded to survey questions covering health, family, and reproductive topics. Facility data were also collected from the six sites. The goal was to undertake a census of facilities that offer FP based on a master list of health facilities and pharmacies. Data were collected from 205 (of 269) health facilities including public and private hospitals, health centers, and health clinics, as well as facilities run by a nongovernmental or faith‐based organization. Data were also collected from 518 (of 576) pharmacies. At each health facility, a facility audit and provider interviews were undertaken. An audit was undertaken at each pharmacy. Private doctors, that is doctors operating a private clinical practice on their own, were not included in the study sample because less than 2% of women in our data report receiving contraceptives from a private doctor, nurse, midwife, healer, or nonmedical shop.

The multistage sampling design allows the location of a woman's home to be approximated by the centroid of the primary sampling unit from which she is drawn. The measured distance between a woman's home and a facility is measured as the straight‐line distance using ArcGIS software. Further details on the multistage sampling design and missing facilities are provided in the Supporting Information, Section A.

### 3.2 Variables

Analysis focuses on two dependent variables: ideal number of children and usage of any FP method. The former is determined by a woman's response to the survey question “If you could have exactly the number of children you wanted to have in your whole life, how many would that be?” 3Ashraf, Field, and Lee (2014) highlight the importance of husband's preferences in FP decisions. In our data, a husband's preference for additional children is reported by the wife. As such, we use the wife's preferred number of children only in our empirical analysis. Empirically, we find a high degree of correlation between the preferences of the wife and the wife's report of her husband's preferences. Table 1 reveals that 18.2% of the sample responds that the ideal number is up to God and 3.4% either does not answer or provides some other non‐numeric response. For those providing a numeric response, the mean and median ideal number of children are 4.74 and 4, respectively. 4Women also report their actual number of children, of which the sample mean is 1.96. Table 1 also reports the proportion of women reporting no method, modern method (i.e., implant, IUCD, injection, pill, morning after pill, condom, spermicide, and/or sterilization), and traditional method (i.e., calendar method, withdrawal, and/or lactational amenorrhea). Few women report using traditional methods (1.5%); thus, estimation focuses on use of *any* FP method (16.6%). 5Of the 1,596 women using FP methods, 220 (13.8%) use a long‐term modern method (e.g., male or female sterilization, an implant, or IUD), 1,035 (64.9%) use a short‐term anticipatory modern method (e.g., pill or injection), 196 (12.3%) use a short‐term non‐anticipatory modern method (e.g., male or female condom, spermicide, or morning after pill), and 145 (9.0%) use a traditional method. In Table S3, we present results for models after (a) dropping traditional method users (column 2) and (b) recoding traditional method users as nonusers (column 3). Results are robust to these specifications.


**Table 1:** Summary of dependent variables

	Frequency	Percentage (%)
Ideal Number of Kids		
0	8	0.1
1	15	0.2
2	349	3.6
3	996	10.4
4	2576	26.8
5	1739	18.1
6	1030	10.7
7–8	550	5.7
9–10	221	2.3
11+^a^	51	0.5
“Up to God”	1749	18.2
Other non‐numeric or missing response	330	3.4
Total	9614	100.0
Family planning method^b^		
None	7997	83.2
Traditional	145	1.5
Modern	1451	15.1
No response	21	0.2
Total	9614	100.0

a The largest reported number of ideal kids is 20. In estimation, the range is capped at 15.

b There are 20 women that report using both modern and traditional methods; they are grouped with the former. Pregnant women (601) are not asked the family planning question; they are coded as using no family planning methods. Of the 181 women who cannot become pregnant, 11 do not answer the family planning question; they are coded as using no family planning methods.

To determine the best way to define a woman's health service environment (HSE), we estimated our joint model using a 0.5, 1, and 2‐kilometer radius around the centroid of the respondent's primary sampling unit using Global Position System coordinates to calculate the distances. The 1‐km radius specification produced the strongest facility effects, as well as the largest likelihood function value. The results from each of these alternative specifications are presented in Tables S5 and S6. Data from health facilities were collected using a facility audit, provider interviews, and exit interviews; exit interviews were only collected from larger facilities and are not included in this analysis. The facility audit obtained information on services offered, types of providers, FP methods available, stock outs, cost of methods, and equipment in the facility. The provider survey asked about the provider's training, methods offered, biases towards methods, and experiences with integrated services. For all pharmacies, a pharmacy audit obtained information on staffing, methods available, stock outs, cost of methods, and equipment.

Quality of FP has often been defined by six areas: (a) choice of methods; (b) information given to clients; (c) technical competence; (d) interpersonal relations; (d) follow‐up/continuity mechanisms; and (e) appropriate constellation of services (Bruce, 1990). The facility‐level questionnaires used for this study were designed with these elements in mind. We include measures from a number of these areas in our analysis—for example, number of methods offered (Area 1); information, education, communication materials, and facility outreach (Area 2); number of doctors, midwives, etc. (Area 3); and advice during non‐FP visits and other services offered at facility (Area 6). Measurement of interpersonal relations often requires observations of client‐provider relations, whereas follow‐up/continuity mechanisms generally require information from clients that were not available from all facilities; thus, quality measures from these areas are not included.

Table 2 reports the mean and standard deviation of these variables at the HSE and individual level. 6Our decision to aggregate across facilities was guided by a recent survey paper that looked at the determinants of modern contraceptive use and notes that “ensuring a wide range of affordable contraceptive methods are offered, making services widely accessible through multiple service delivery channels” is the most important factor in increasing contraception use (Mwaikambo, Speizer, Schurmann, Morgan, & Fikree, 2011). This seems to suggest that a saturation of services may be more important than simply having any services available. Several variables require explanation. A high‐volume facility was defined by the program as a facility that covers a catchment area with a large population, has a large number of clients, provides a full range of FP methods, and employs trained personnel for FP and reproductive health services (MLE, ISSU, 2012b). The facility audit allows health facilities to report the use of up to eight information, education, and communication FP tools (e.g., posters, brochures, and demonstration models), which were visually verified by the survey administrator. Using information in the provider survey, we identify facilities that have “standards or protocols for FP services” and facilities that regularly provide FP information during a non‐FP consultation. For each of these variables, we calculate the proportion of providers within each facility responding “yes,” and then aggregate up to the HSE level by averaging across all facilities within the 1‐km buffer. A “social program” offers methods at a discounted price. These programs are not very common because most FP methods are offered in the public sector at low or no cost. The last variable indicates that all health facilities and pharmacies within the HSE failed to participate in the survey. 7There are 32 health facilities and 37 pharmacies that did not participate in the survey. The number of public facilities, private facilities, and pharmacies is calculated including these nonresponding facilities. All other variables are calculated only using facilities participating in the survey. There are six HSEs for which only nonparticipating facilities are observed.


**Table 2:** Summary of family planning access and quality variables

	HSE level		Individual level	
	Mean	*SD*	Mean	*SD*
Health facility variables				
Number of public facilities	3.16	2.16	2.93	2.13
Number of private facilities	0.97	1.23	0.86	1.14
Number of high volume facilities	1.34	1.50	1.27	1.46
Number with (infant) delivery services	1.90	1.52	1.83	1.48
Average number of doctors in facility	0.81	1.38	0.74	1.29
Average number of nurses in facility	2.60	3.31	2.38	3.12
Average number of midwives in facility	2.16	2.14	2.01	1.98
Average number of FP methods sold	4.45	1.72	4.36	1.80
Average number IEC FP tools	2.15	1.13	2.08	1.13
Proportion with family planning protocol	0.86		0.85	
Proportion providing FP info during non‐FP visit	0.74		0.75	
Any facility has a health social worker	0.43		0.39	
Any facility has a FP outreach program	0.80		0.77	
Any facility gives FP talks to community	0.87		0.86	
Pharmacy variables				
Number of pharmacies	10.17	6.71	9.26	6.21
Average number of methods sold	4.40	0.92	4.42	0.93
Proportion with multiple pharmacists	0.22		0.19	
Proportion requiring trained FP consultant	0.45		0.48	
Proportion where staff can advise on FP	0.75		0.73	
No health facility or pharmacy information	0.02		0.02	
Observations^a^	263		9,263	

*Notes*. IEC = information, education, and communication; FP = family planning, HSE = health service environment.

a The table reports average HSE characteristics across both HSEs and individuals. For example, for *number of public facilities*, we first calculate total number of facilities within each of the 263 HSEs. In column 1, we report the average of these counts across the 263 HSEs. In column 3, we define the number of public facilities corresponding to each individual as the number within their particular HSE, then take the average across individuals. Other variables are defined similarly.

Table 2 reveals that the average health facility and pharmacy are similar in the number of FP methods offered and are equally likely to have a social program. The average health facility reports significant efforts to promote FP practices, as 86% have a FP protocol, 74% provide FP information during non‐FP consultations, 80% have information, education, and communication outreach programs, and 87% host health talks for the community.

Table 3 describes individual‐level variables used in the analysis. We generate measures of both household income and wealth. We measure income by aggregating and transforming household consumption information into daily individual World Bank International Purchasing Power Parity Dollars (2005). The consumption information contributing to the measure includes food staples such as proteins, vegetables, and drinks, as well as items to support the household including gas, electricity, and transport. The wealth quintile calculations were done using the methodology used by the Demographic and Health Survey data sets. 8See: http://dhsprogram.com/topics/wealth‐index/Wealth‐Index‐Construction.cfm. The survey gathered information on household assets including whether the household has water, electricity, car, bicycle, and numerous other items that urban households may possess. The wealth index was calculated using principal components analysis. Quintiles are formed from surveyed households in the six study cities. Table 3 contains descriptive statistics of the income measure only because, by definition, 20% of the sample is in each wealth quintile. 9Given that the wealth measure was produced via principal components analysis, the cardinality of the measure cannot be clearly interpreted. However, by looking at average daily consumption within each wealth quintile, one can gain some understanding of the purchasing power of each group. Starting with the poorest quintile, mean daily purchasing power dollars are 3.09 (s.d. 2.32), 3.38 (s.d. 3.06), 3.43 (s.d. 4.20), 3.97 (s.d. 2.98), and 5.12 (s.d. 3.82). The average individual lives on $3.81 a day; however, we find that the income measure has no predictive power, so the results presented below control for wealth quintile only. There are six primary ethnic groups in Senegal (i.e., Wolof, Poular, Serer, Diola, Mandingue, and Soninke), though the three largest (Wolof, Poular, and Serer) make up 81% of the sample. We classify a woman as working if she reports “performing some job for which she is paid in cash or kind” in the last 7 days. We classify both married and cohabitating women as “married,” though only 23 of the 9,614 women interviewed report cohabitation.

**Table 3:** Summary of individual level independent variables

Variable	Mean	*SD*
Age	27.80	9.06
Education		
None	0.35	
Primary school	0.34	
Middle school	0.19	
High school or higher	0.12	
Income	3.81	3.45
Muslim	0.95	
Ethnicity		
Wolof	0.41	
Poular	0.21	
Serer	0.19	
Other	0.19	
Worked in last week	0.36	
Listens to radio	0.74	
Reads newspaper/magazine	0.32	
Has personal cell phone	0.72	
Has internet access	0.13	
Number of beds in the home	4.65	2.72
Employs help in the home	0.22	
Running water in the home	0.34	
Toilet in the home	0.87	
Married^a^	0.55	
Partner: Other wives	0.30	
Partner: Age	34.16	18.97
Partner: Education		
None	0.40	
Primary school	0.11	
Middle school	0.08	
High school or higher	0.14	
Educated but unsure of grade	0.29	
Partner: works	0.91	
Observations^b^	9,263	

a Mean and standard deviation for all variables below “married” are calculated for married individuals only. In estimation, partner variables are coded as zero for unmarried women.

b The sample is reduced from 9,614 to 9,263 because sample inclusion requires that women provide a numeric or “up to God” response to the ideal children question (−330), answer the family planning usage question (−20), and report their marriage status (−1). The characteristics of nonresponders are presented in Table S4. Other observations with missing variable values are replaced with sample means. The number of missing observations by variable is as follows: number of beds in home (11), employ help in home (16), running water in home (3), other wives (55), partner works (57), worked last week (12), listens to radio (8), reads newspaper/magazine (9), has personal cell phone (16), and has internet access (20).

### 3.3 Geographic variation in contraceptive use

**Figure 1:**
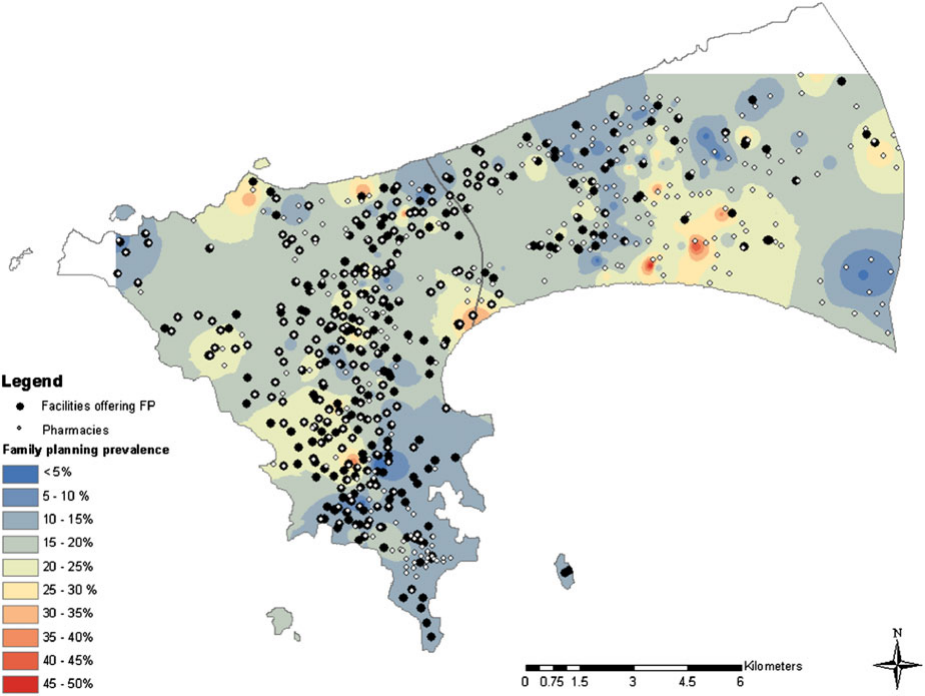
Percentage of women using family planning methods by location of home, Dakar region. *Notes*. We used inverse distance weighting to create a smoothed surface of the proportion of women in individual clusters using FP methods. This strategy allowed us to visualize FP prevalence spatially without revealing the location of individual sampled clusters, as well as providing an estimate of FP prevalence in unsampled areas, based on prevalence in nearby clusters and the distance to those clusters. Interpolation was done in ArcMap v. 10.4. FP = family planning.

Figure 1 is a map of the four districts of the Dakar region of Senegal that are included in our sample (Dakar, Guédiawaye, Pikine, and Mbao). The map shows both the location of all health facilities that provide FP care and an interpolated surface representing the proportion of women living in an area that uses FP methods. This figure motivates our empirical analysis in two ways: First, the map suggests that areas where FP is most popular also tend to be densely populated with health facilities and pharmacies whereas areas void of facilities display lower FP use. This suggests that, even in urban environments, access may play an important role. Second, some of the areas where FP use is lowest are also densely populated with facilities. This finding suggests that quality of facilities, in addition to access, might play an important role. 10Alternatively, this finding could suggest that there are simply geographic differences in FP practices that are unrelated to health facility access or quality, which motivates the use of district level fixed effects. Our analytic approach, therefore, uses multivariate methods, controlling for endogenous facility placement, to determine the relative importance of facility access and quality in determining urban contraceptive use.

### 3.4 Main estimation results

In our preferred specification, we estimate the up to God, ideal number of children, and contraceptive use equations jointly. We follow Mroz's (1999) upward testing approach; adding points of support to the heterogeneity distribution until the likelihood function improvement from an additional point is no longer significant. We find four points of support to be sufficient. The mass points for the heterogeneity distribution are presented in Table 4. A Wald test of the null hypothesis that the heterogeneity parameters are jointly equal to zero is rejected at a 1% level of significance. This suggests that the up to God and ideal number of children responses are endogenous to the contraceptive use decision; thus, we focus on results for the random effects model throughout. Results for the uncorrelated model are presented for comparison. 11We have also conducted our analysis using a measure of “additional kids wanted” in place of “ideal number of children.” Our main findings from this analysis are consistent with the results presented and can be found Table S7.


**Table 4:** Unobserved heterogeneity distribution

Points of support in discrete distribution					
	1 (Simple)	2	3	4	5
Number of parameters	259	263	267	271	275
Likelihood function value	−21538.74	−20707.22	−20689.71	−20677.25	−20675.73
Likelihood function improvement		831.52	17.51	12.46	1.52
Probability Weights					
1	1	0.96	0.63	0.49	0.12
2		0.04	0.04	0.04	0.04
3			0.32	0.35	0.35
4				0.11	0.11
5					0.37

Parameter estimates are presented in Tables 5, 6, and 7. Of key interest in this research is the effect that local health facility characteristics have on FP use. Results suggest that measures of health facility access within 1 km of a woman's home have little effect. As shown in Table 5, neither the number of public and private facilities nor pharmacies has any significant effect on the likelihood that a woman uses FP. This finding is not surprising given the urban setting. In our sample, 98.75% of women have at least one health facility or pharmacy within a kilometer of their home. The average health facility/pharmacy carries 4.4 FP methods, has few stock outages, and prices for contraceptives are very low; therefore, the marginal increase in access to contraceptives provided by one more facility is minimal. The quality of facilities near a woman's home has a more significant impact on her FP decisions. Contraceptive use significantly increases with the proportion of pharmacies within 1 km of a woman's home that have multiple pharmacists and decreases with the proportion of these pharmacies allowing nonpharmacist staff to advise on FP issues. We also find that FP protocols in health facilities significantly increase the likelihood that women use FP. 12The finding that family planning protocols increase usage is specific to the survey's definition of a protocol. The information is taken from the facility provider interviews; providers were asked “are there any standards or protocols on family planning services?” The survey does not ask about the details of these protocols; however, they are generally adopted from international agencies such as the World Health Organization. See: http://www.who.int/reproductivehealth/publications/family_planning/9780978856304/en/
 Having a health social worker within 1 km does not significantly alter the likelihood that a woman uses FP, but it decreases her ideal number of children.

**Table 5:** Results for any use of family planning methods

Variable	Simple model			Random effects		
	Coef.		*SE*	Coef.		*SE*
Health facility and pharmacy variables						
Number of public facilities	0.015		0.047	−0.005		0.079
Number of private facilities	−0.015		0.060	−0.053		0.108
Number of pharmacies	−0.009		0.062	0.044		0.109
Number of high volume facilities	−0.008		0.015	−0.028		0.026
No facilities/pharmacies participate in survey	−0.218		0.331	−0.317		0.545
Average number of doctors at facility	−0.095		0.121	−0.136		0.209
Average number of nurses at facility	0.021		0.040	0.086		0.072
Average number of midwives at facility	0.006		0.070	−0.075		0.122
Proportion of pharmacies with multiple pharmacists	0.889**		0.433	1.924***		0.752
Proportion of facilities with a family planning protocol	0.285		0.257	0.729*		0.442
Proportion of pharmacies requiring FP training	−0.215		0.247	−0.504		0.445
Proportion of pharmacies allowing staff to advise on FP	−0.176		0.241	−0.763*		0.459
Average FP methods sold at health facility	−0.012		0.052	−0.028		0.092
Average FP methods sold at pharmacy	0.059		0.067	0.174		0.128
Any facility has FP social program	−0.051		0.131	0.206		0.245
Any pharmacy has FP social program	0.115		0.163	0.328		0.281
Any facility has a health social worker	0.179		0.147	0.153		0.264
Some facility has community outreach program	−0.123		0.129	−0.188		0.234
Some facility conducts community talks on FP	−0.100		0.202	−0.474		0.360
Proportion giving FP advice during non‐FP visit	−0.041		0.294	0.340		0.538
Average number if IEC materials at facility	0.041		0.063	0.014		0.113
Individual variables						
Ideal number of kids	−0.178***		0.065	−0.239**		0.096
Number of kids is left “up to God”	−1.155***		0.388	−1.389		1.058
Ideal number of kids (married)	0.130*		0.068	−0.130		0.117
Number of kids is left “up to God” (married)	0.518		0.410	−1.714**		0.863
Age (reference 40+)						
15–19	−2.120***		0.211	−2.884***		0.334
20–24	−0.695***		0.129	−1.179***		0.237
25–29	−0.427***		0.116	−0.709***		0.208
30–34	−0.043		0.106	−0.070		0.217
35–39	0.054		0.105	0.172		0.209
Highest level of education (reference: none)						
Primary school	0.339***		0.078	0.499***		0.177
Middle school	0.613***		0.118	1.026***		0.283
High school or higher	0.433***		0.145	0.455		0.314
Ethnicity (reference: other)						
Wolof	−0.076		0.090	−0.237		0.180
Poular	−0.036		0.100	−0.237		0.194
Serer	−0.139		0.101	−0.307		0.192
Socioeconomic status (reference: 1st quintile)						
2nd quintile	0.078		0.103	0.047		0.182
3rd quintile	0.067		0.103	0.079		0.183
4th quintile	0.006		0.107	‐0.082		0.183
5th quintile	−0.138		0.114	−0.351*		0.195
Muslim	−0.324**		0.145	−0.886***		0.291
Worked last week	0.241***		0.065	0.357***		0.122
Listens to radio	0.002		0.072	0.023		0.128
Reads the newspaper/magazines	−0.074		0.091	−0.194		0.166
Has a cell phone	0.005		0.078	0.116		0.132
Has internet access	−0.144		0.128	−0.094		0.224
Married	1.660***		0.393	3.960***		0.796
Partner: Has other wives	−0.195**		0.080	−0.213		0.191
Partner: Age	−0.120**		0.049	−0.158*		0.094
Partner: Age is not known	−0.800***		0.230	−1.122**		0.475
Partner: Highest education (reference: none)						
Primary school	0.475***		0.114	1.108***		0.344
Middle school	0.155		0.133	0.413		0.376
High school or higher	0.281**		0.115	0.600**		0.251
Educated but unsure of grade	0.253***		0.088	0.448***		0.174
Partner works	0.050		0.119	0.197		0.234
Constant	−2.272***		0.637	‐9.628***		1.769
DFRE variables						
Point 1 (normalized to zero)				0.000		0.000
Point 2				9.038***		1.789
Point 3				9.503***		1.725
Point 4				8.381***		1.929
District fixed effects	Yes			Yes		
Observations	9,263			9,263		

*Notes*. IEC = information, education, and communication; FP = family planning.

* Data statistically significant at the 10% level.

** Data statistically significant at the 5% level.

*** Data statistically significant at the 1% level.

**Table 6:** Results for ideal number of kids

Variable	Simple model			Random effects		
	Coef.		*SE*	Coef.		*SE*
Health facility and pharmacy variables						
Any facility has a health social worker	−0.186**		0.072	−0.160***		0.058
Any facility has comm. outreach program	0.099		0.067	0.055		0.052
Any facility conducts community talks on FP	−0.087		0.101	−0.046		0.086
Proportion giving FP advice during non‐FP visit	0.089		0.140	0.148		0.116
Average number if IEC FP materials at facility	−0.026		0.024	−0.035*		0.020
Number of health facilities with delivery services	0.034		0.023	0.024		0.019
Individual variables						
Age (reference: 40+)						
15–19	−0.040		0.088	0.142*		0.077
20–24	−0.011		0.083	0.185**		0.074
25–29	−0.029		0.081	0.156**		0.072
30–34	−0.108		0.079	0.067		0.072
35–39	−0.071		0.081	0.071		0.074
Highest level of education (reference: none)						
Primary school	−0.477***		0.050	−0.319***		0.043
Middle school	−0.674***		0.069	−0.431***		0.056
High school or higher	−0.752***		0.084	−0.524***		0.065
Ethnicity (reference: other)						
Wolof	0.030		0.056	0.027		0.046
Poular	−0.098		0.062	−0.077		0.051
Serer	0.102*		0.062	0.039		0.049
Socioeconomic status (reference: 1st quintile)						
2nd quintile	−0.107*		0.064	−0.046		0.055
3rd quintile	−0.132**		0.067	−0.086		0.055
4th quintile	−0.211***		0.073	−0.131**		0.059
5th quintile	−0.205**		0.084	−0.132**		0.067
Muslim	0.460***		0.086	0.349***		0.061
Worked last week	0.040		0.042	0.027		0.035
Listens to radio	−0.027		0.045	0.050		0.038
Reads the newspaper/magazines	−0.103*		0.054	−0.093**		0.042
Has a cell phone	0.019		0.045	0.026		0.039
Has internet access	−0.127**		0.063	−0.154***		0.047
Married	0.298		0.183	0.213		0.167
Partner: Other wives	−0.036		0.063	−0.076		0.056
Partner: Age	0.051		0.035	0.057**		0.033
Partner: Age is not known	0.156		0.161	0.252**		0.146
Partner: highest education (reference: none)						
Primary school	−0.083		0.089	−0.088		0.076
Middle school	−0.164*		0.099	−0.057		0.079
High school or higher	−0.130		0.086	−0.116		0.073
Educated but unsure of grade	−0.258***		0.067	−0.219***		0.059
Partner works	−0.061		0.092	−0.043		0.083
Number of beds in the home	0.041***		0.009	0.031***		0.007
Employs help in the home	−0.058		0.055	−0.049		0.043
Running water in the home	0.022		0.043	0.048		0.035
Toilet in the home	−0.144**		0.063	−0.146***		0.051
Constant	4.324***		0.237	4.137***		0.137
DFRE variables						
Point 1 (Normalized to Zero)				0.000		0.000
Point 2				4.823***		0.128
Point 3				0.122		0.096
Point 4				−0.128		0.090
District fixed effects	Yes			Yes		
Observations	7519			l7519		

*Notes*. IEC = information, education, and communication; FP = family planning.

* statistically significant at the 10% level.

** statistically significant at the 5% level.

*** statistically significant at the 1% level.

**Table 7:** Results for “up to God”

Variable	Simple model			Random effects		
	Coef.		*SE*	Coef.		*SE*
Health facility and pharmacy variables						
Any facility has a health social worker	−0.474***		0.110	−0.751***		0.227
Any facility has comm. outreach program	0.080		0.094	0.203		0.160
Any facility conducts community talks on FP	−0.190		0.142	−0.286		0.208
Proportion giving FP advice during non‐FP visit	0.863***		0.207	1.302***		0.398
Average number if IEC FP materials at facility	−0.107***		0.037	−0.169**		0.069
Number of health facilities with delivery services	0.155***		0.034	0.217***		0.058
Individual variables						
Age (reference: 40+)						
15–19	−0.432***		0.122	−0.692***		0.219
20–24	−0.476***		0.114	−0.757***		0.199
25‐29	−0.243**		0.107	−0.419***		0.163
30–34	−0.567***		0.108	−0.864***		0.202
35–39	−0.447***		0.108	−0.721***		0.194
Highest level of education (reference: none)						
Primary school	−0.357***		0.069	−0.552***		0.139
Middle school	−0.629***		0.112	−1.069***		0.335
High school or higher	−0.659***		0.155	−1.085***		0.340
Ethnicity (reference: other)						
Wolof	0.125		0.088	0.202		0.140
Poular	0.119		0.097	0.143		0.147
Serer	−0.033		0.101	−0.038		0.154
Socioeconomic status (reference: 1st quintile)						
2nd quintile	0.094		0.091	0.068		0.135
3rd quintile	0.024		0.097	0.014		0.145
4th quintile	−0.121		0.110	−0.244		0.177
5th quintile	−0.069		0.129	−0.135		0.200
Muslim	0.610***		0.191	1.033***		0.349
Worked last week	−0.010		0.062	−0.032		0.095
Listens to radio	−0.395***		0.063	−0.597***		0.136
Reads the newspaper/magazines	−0.267***		0.093	−0.489**		0.203
Has a cell phone	0.108*		0.065	0.152		0.095
Has internet access	−0.745***		0.147	−1.200***		0.345
Married	−0.489*		0.254	−0.665*		0.376
Partner: other wives	0.165*		0.080	0.225*		0.123
Partner: age	0.146***		0.047	0.200***		0.071
Partner: age is not known	0.923***		0.219	1.254***		0.351
Partner: highest education (reference: none)						
Primary school	−0.257**		0.132	−0.371*		0.214
Middle school	−0.860***		0.179	−1.291***		0.342
High school or higher	−0.133		0.130	−0.200		0.209
Educated but unsure of grade	−0.123		0.085	−0.189		0.125
Partner: works	0.210*		0.122	0.301*		0.175
Number of beds in the home	0.018		0.013	0.032		0.020
Employs help in the home	−0.149		0.091	−0.249*		0.144
Running water in the home	−0.005		0.067	0.001		0.101
Toilet in the home	0.366***		0.099	0.570***		0.201
Constant	−0.800**		0.354	−3.082***		0.702
DFRE variables						
Point 1 (normalized to zero)				0.000		0.000
Point 2				−10.923***		0.451
Point 3				0.238		0.595
Point 4				4.371***		0.959
District fixed effects	Yes			Yes		
Observations	9,263			9,263		

*Notes*. IEC = information, education, and communication; FP = family planning.

* Data statistically significant at the 10% level.

** Data statistically significant at the 5% level.

*** Data statistically significant at the 1% level.

Coefficients on the individual‐level independent variables are generally of the hypothesized sign and significance in each of the three equations. For example, we find that the likelihood of using FP increases with age. We also find that more education leads to significantly higher FP usage, lower ideal number of children, and lower likelihood of responding up to God. Muslims are significantly less likely to use FP methods, idealize more children, and are more likely to respond up to God. Married women are much more likely to use FP methods than unmarried women, as unmarried sex is uncommon in Senegal. 1396% of the unmarried women in our data report that they have not had sex in the last three months. Furthermore, the likelihood of FP use is significantly higher for women with educated husbands, whereas ideal number of children is lower. In general, married women are less likely to respond up to God to the ideal number of children question, though the likelihood of this response is higher for women in polygynous marriages, for women with older husbands or husbands with unknown ages, and for women with uneducated husbands. Our results suggest that families in higher wealth quintiles desire fewer children; however, after controlling for ideal family size, those in the highest wealth quintiles are less likely to use FP than those in the first quintile.

The coefficients on the up to God and ideal number of children variables in the FP equation have the expected sign and significance. Women who idealize a greater number of children are less likely to use FP than those wanting fewer children. This negative relationship is intensified for married women, who have greater resources to both meet and support their desire for additional children. FP use is also lower for women responding that they wish to leave their number of children up to God than women wanting few children. Again, this negative relationship is intensified for married women. According to these estimates, a married woman responding up to God to the ideal children question uses FP methods with equal probability to an identical married woman desiring 8.4 children.

### 3.5 Model specification tests

We conducted specification tests on our model. We briefly summarize these tests here and provide greater detail in the Supporting Information, Section B.

One concern is endogenous program targeting or that health facilities in low usage areas may increase their FP outreach, training, etc. in an effort to improve usage. To address this concern, we include district‐level fixed effects in our model. We test for program targeting by (a) estimating our model with and without these fixed effects, (b) calculating the joint covariance matrix (see Mroz, 1987 for details) of the two sets of estimated coefficients, and (c) conducting a Wald test for significantly different effects of the independent variables. We find a small, statistically significant difference in the estimates, suggesting strategic program targeting, therefore, justifying our inclusion of fixed effects.

A second concern is the identification of the model parameters. Although the model is identified off nonlinearities alone, we utilized exclusion restrictions to improve efficiency. We conducted several tests to show that (a) these variables have a jointly significant effect on the up to God and ideal number of children variables, (b) these variables have no direct effect on the FP decision, and (c) model parameters are quite stable when we rely solely on the nonlinearity of the model for identification, which is consistent with Mroz's (1999) Monte Carlo study on identification using discrete factor models.

A final concern is whether the up to God and ideal number of children variables are endogenous. We estimate the model both with and without correlation between the model's unobservables. These two models reveal statistically significant and important differences in the point estimates of the ideal number of children equation, suggesting endogenous selection into a numeric response. The two models also reveal differences for the FP equation; however, because the two models make different assumptions about the error variance, we cannot statistically compare point estimates from the two estimation procedures. Therefore, we use simulations to test for differences between the models. These simulations provide clear evidence that the response to the ideal number of children question is endogenous to the FP decision.

### 3.6 Simulations

Simulations are done using a parametric bootstrap method that takes advantage of the fact that, under general conditions, maximum likelihood estimators for the coefficients are asymptotically normally distributed. Thus, all simulations use the estimated covariance matrix to sample from a multivariate normal distribution centered at the point estimates of the coefficients. The marginal effects and standard errors are calculated using 1,000 bootstrap replications.

Our simulations are motivated by policy efforts to reduce family sizes in Senegal and in other sub‐Saharan and West African countries. Senegal is part of the Ouagadougou Partnership of French speaking African countries who have joined together to support FP at the country and regional levels. In February of 2011, the countries issued a call to action that had seven points, one of which was to increase by 30% the number of health professionals capable and authorized to offer a range of FP/RH services. 14The full list of action items can be seen at http://www.prb.org/Publications/Reports/2012/ouagadougou‐partnership‐en.aspx. Specific to Senegal, a Gates funded project: Initiative Sénégalaise de Santé Urbaine (ISSU), also initiated in 2011, began both demand and supply side activities to promote FP. Working in both public and private sectors, the project sought to strengthen pharmacies and provide logistical support of FP supplies. Demand side activities included mass media, religious leader, and interpersonal communications (see Speizer et al., 2017).

We use the model to predict the change in contraceptive use (and other endogenous variables) when variables that measure health facility quality are altered. For example, the first row of Table 8 shows the effect of ensuring that all women have at least one health social worker within 1 km of their home. This change decreases the probability of responding up to God by 6.5 percentage points and, conditional on answering the question, decreases the average ideal number of children by 0.167. Furthermore, the combined direct effect of the additional health social worker, along with the effect of the altered endogenous variables, is a 1.8 percentage point increase in the likelihood of using a FP method. All of these effects are statistically different from zero at a 1% level of significance.

**Table 8:** Simulated changes in endogenous variables

Change in independent variable	Up to God			Ideal kids			Family planning use		
	Change		*SE*	Change		*SE*	Change		*SE*
Any health social worker: 0 to 1	−0.065***		0.0005	−0.167***		0.0018	0.018***		0.0005
Health facilities have family planning protocol: 0% to 50%							0.021***		0.0004
Health facilities have family planning protocol: 50% to 100%							0.020***		0.0004
Pharmacies have multiple pharmacists: 0% to 50%							0.059***		0.0007
Pharmacies have multiple pharmacists: 50% to 100%							0.060***		0.0007
Education: None to primary	−0.054***		0.0004	−0.331***		0.0013	0.039***		0.0003
Education: Primary to middle	−0.040***		0.0005	−0.121***		0.0014	0.038***		0.0004
Education: Middle to secondary	−0.001*		0.0006	−0.093***		0.0016	−0.034***		0.0004
Ideal kids: 5 to 3							0.037***		0.0004

*Note*. For up to God and family planning use, the table reports the predicted *percentage point* change in the likelihood of a positive response. For ideal number of kids, the table reports the predicted change in the number of kids.

* Data statistically significant at the 10% level.

** Data statistically significant at the 5% level.

*** Data statistically significant at the 1% level.

Our simulations suggest that, among the policy variables we study, instituting FP protocols and increasing the number of pharmacists are the most effective methods for increasing contraceptive use. Increasing the proportion of facilities with an FP protocol from 50% to 100% increases contraceptive use by 2.0 percentage points. Moreover, shifting the proportion of pharmacies with multiple pharmacists from 0% to 50% would increase FP method use by 5.9 percentage points. 15Currently, 85% of health facilities in our sample have a FP protocol and 22% of the pharmacies have multiple pharmacists. Policy makers may wish to know how FP decisions within Senegal would respond to reasonable changes in these levels. As such, we have also simulated the response to (a) an increase the proportion of health facilities with an FP protocol from 85% to 100% and (b) an increase in the proportion of pharmacies employing multiple pharmacists from 22% to 50%. We find that FP use increases by 0.6 (s.e. 0.01) and 3.4 (s.e. 0.04) percentage points, respectively. Importantly, in these simulations we assume that manipulated facility quality characteristics are uniform across HSEs, as our model does not suggest which HSEs would be most responsive to any particular policy. To our knowledge, this is the first paper to quantify the important role that pharmacists play in promoting FP practices. The effects of increasing education levels and simulating a reduction in ideal family size, possibly through media campaigns, are also reported in Table 8.

## 4 CONCLUSION

We have three main findings. First, access to health facilities and contraceptive methods is not lacking for Senegalese women living in urban environments; therefore, increasing the number of health facilities, number of pharmacies, or average number of contraceptive methods offered at facilities and pharmacies would not increase FP use. However, we do find that the quality of facilities and pharmacies affect FP practices. Second, we find that a woman's reported ideal number of children both affects and is endogenous to her usage of contraceptives. Failure to control for this endogeneity leads to bias in estimated marginal effects. Third, understanding the preferences of women reporting that they wish to leave their total number of children up to God is important for programs and policies aimed at reducing family size and increasing FP use. In our data, these women are among the least likely to use FP and have (on average) 0.87 more children than the women providing a numerical response. Given these results, we would advise against the popular practice of dropping up to God responders.

Our findings have program and policy implications that can be used to help the government of Senegal attain their national‐level FP goals. These goals included increasing the contraceptive prevalence rate among married women nationally from 12% (in 2010) to 30%. Our results suggest that access to qualified pharmacists is a particularly important determinant of FP use. For example, a woman living in an area where all pharmacies within a 1‐km radius (a) have multiple pharmacists and (b) do not allow untrained staff to advise on FP methods is roughly two times more likely to use FP than a woman living in an area without these quality indicators. Moreover, we find that FP use is higher in areas where facilities have FP protocols and that women desire fewer children in areas with health social workers. We also find a strong, positive relationship between education and FP use. That said, improving educational outcomes is likely the most expensive and difficult way to address low FP use in the short run. Thus, in order for Senegal to attain their national‐level FP goals, we recommend that program managers and policy makers consider: (a) supporting health facilities to hire health social workers and create FP protocols and (b) encouraging pharmacies to employ more actual pharmacists, rather than untrained staff. Strengthening education programs serves as a more long‐run policy objective, given the strong effects not only on FP use but also on infant and maternal health.

## Supporting information

Table S1. Results with No District Fixed Effects
Table S2. Family Planning Use, Including Exclusion Restrictions
Table S3. Results for Any Use of Family Planning Methods; Different Handling of Traditional Method Users
Table S4. Individual‐Level Independent Variables, Non‐Responders
Table S5. Results with Health Service Area Defined as 0.5 KM Radius Around PSU Centroid
Table S6. Results with Health Service Area Defined as 2 KM Radius Around PSU Centroid
Table S7. Results with Additional Kid Wanted
