# Supplementary material for: The effects of health facility access and quality on family planning decisions in urban Senegal
Source: Health Econ. 2017 Nov 2;27(3):576–91. doi: 10.1002/hec.3615 (PMC5867202; doi:10.1002/hec.3615)
Supplement: Supplementary file 1 — Table S1. Results with No District Fixed Effects Table S2. Family Planning Use, Including Exclusion Restrictions Table S3. Results for Any Use of Family Planning Methods; Different Handling of Traditional Method Users Table S4. Individual‐Level Independent Variables, Non‐Responders Table S5. Results with Health Service Area Defined as 0.5 KM Radius Around PSU Centroid Table S6. Results with Health Service Area Defined as 2 KM Radius Around PSU Centroid Table S7. Results with Additional Kid Wanted [file HEC-27-576-s001.docx]

**The Effects of Health Facility Access and Quality on Family Planning Decisions in Urban Senegal**

**Web Appendix**

**A. Survey Details**

Data for this study come from baseline household and facility data collected in 2011 by the Measurement, Learning & Evaluation (MLE) project for the evaluation of the Initiative Sénégalaise de Santé Urbaine (ISSU) in Senegal. Data were collected from six urban sites: Dakar, Guédiawaye, Pikine, Mbao, Mbour, and Kaolack. In each site, a multi-stage sampling design was used to select a representative sample of women ages 15-49. In the first stage, using information from the 2002 census classification of primary sampling units (PSU) or clusters (updated in 2009), 32-64 PSUs were selected with probability proportional to population size of the urban site. Prior to selection, PSUs were classified as poor or non-poor based on neighborhood characteristics; the list of characteristics was based on UN-HABITAT (2003) classifications. Half of the selected PSUs in each site were classified as poor, which permitted oversampling of poor women; weights were adjusted so that the data were representative of the sites in the descriptive analyses. In the second stage, a random sample of 21 households was selected from each PSU and all women aged 15-49 from selected households were eligible for interview. Eligible women were asked to give written consent to participate in the study. For eligible women under age 18, the household head was asked for consent to approach the teenager prior to requesting participation and consent. A total of 4950 households were surveyed from 263 primary sampling units. The final sample includes 9614 women from the six urban sites; the response rate was 88.9% (MLE, ISSU, 2012a).

Facility data were also collected from the six sites; this included high volume public and private health facilities as well as lower volume public and private health centers and pharmacies. The goal was to undertake a census of facilities that offer FP based on a master list of health facilities and pharmacies. Of a total of 269 health facilities on the master list, data were collected from 205 of the facilities. Of the 64 facilities not interviewed, 17 were on strike at the time of the survey; 3 were joined with another facility by the time of interview; and the remaining did not offer FP (11), refused (9), were closed/destroyed (18), or the person was not available (6). Of 576 pharmacies on the master list, data were collected from 518. Of the 58 pharmacies not interviewed, 7 were duplicates, 8 were refusals, 14 closed/destroyed/moved, 22 were not found, and 7 were not available at the time of interview. At each health facility, a facility audit and provider interviews (with up to 4 providers per facility depending on the size of the facility) were undertaken. At each pharmacy, a pharmacy audit was undertaken.

**B. Model Specification Tests**

In the paper, we briefly describe a number specification tests performed on our model. These tests are described here in greater detail.

B.1 Endogenous Health Facility Characteristics

The main goal of this research is to determine the effect that local health facility access and quality have on family planning decisions. Estimating a causal effect is complicated by the potential for program targeting. For example, health facilities in areas with low contraceptive usage rates may increase their family planning outreach, training, etc. in an effort to improve usage. Failure to account for program targeting could bias predicted effects. To test for program targeting, we perform preliminary estimations with and without district-level dummies in reduced form models of the up to God, number of ideal children, and family planning usage variables. For each dependent variable, we then calculate the joint covariance matrix (see Mroz, 1987 for details) of the two sets of estimated coefficients (with and without the district dummies) to test whether the inclusion of district-level dummies alters the effects of the independent variables.^[[1]](#footnote-1)^

For the up to God equation, a joint (Wald) test of the null hypothesis that all coefficients are the same with and without district dummies can be rejected at a 1% level of significance. A joint test of the null hypothesis that all of the *health facility* coefficients are the same with and without district dummies is also rejected at a 1% level of significance. We also test these health facility coefficients individually, finding significant differences between the two models only for the coefficients on number of facilities with delivery services and any outreach programs, each at a 1% level.

We perform each of these tests for the models featuring ideal number of children as the dependent variable. None of the tests reveal a significant difference between the coefficients found in the models with and without district dummies.

We also perform these tests for the models featuring family planning usage as the dependent variable. A joint test of the null hypothesis that all coefficients are the same with and without district dummies cannot be rejected, nor can a joint test that all health facility coefficients are the same. We also test each health facility coefficient individually and find three significant differences between the two models. The model without district dummies yields a coefficient of 0.252 on the variable proportion of pharmacies with multiple pharmacists (see Table A below), while the model with district-level dummies yields a coefficient of 0.902 (Table 5). This difference is significant at the 5% level and is consistent with program targeting, as the model without district-level dummies under predicts the positive effect that multiple pharmacists within a pharmacy have on contraceptive use. We also find a significant difference in the coefficients on number of private facilities and any family planning protocol (at the 10% level), though the change in coefficients is not consistent with program targeting.

In total, we find some evidence of endogenous health facility variables due to strategic program targeting; therefore, our final model specification includes district-level fixed effects. We feel that this specification is preferable to treating community unobservables as purely random effects.

B.2 Identification

The family planning equation contains two endogenous right hand side variables: up to God and ideal number of children. The coefficients on these variables are technically separately identified due to their non-linearity (Mroz 1999), though valid exclusion restrictions can improve the efficiency of the estimator. Therefore, we allow home characteristics (i.e., number of bedrooms, presence of running water, indoor toilet, and employing help in the home) and number of facilities with delivery services to enter the up to God and ideal number of children equations, but not the family planning equation. Thus, the model is overidentified on the basis of exclusion restrictions.

To validate these assumptions, we perform several tests (see Angrist and Pischke, 2009 for a detailed discussion of identification in instrumental variables models). First, in order for our exclusion restrictions to be valid, they must have a significant effect in the up to God and ideal number of children equations. Joint tests of the null hypotheses that these five coefficients have no effect on the up to God equation and the ideal number of children equation are each rejected at a 1% level of significance. However, Tables 6 and 7 reveal that the coefficients on the variables employs help in the home and has running water in the home are not individually significant (at a 10% level) in either equation.

A second condition for the validity of the restrictions is that they have no direct effect on contraceptive use. There are two ways to test this assumption. First, we can use the fact that the model is technically identified without exclusion restrictions to verify that the excluded variables do not have direct effects on the family planning decision. To do so, we simply include the excluded variables in the family planning equation. We fail to reject a joint test of the null hypothesis that the five coefficients are equal to zero at a 1% level of significance. Table B (below) reveals further that none of the five exclusion restrictions has an individually significant effect on the family planning decision. In addition, note that even when we include the identifying variables in the family planning equation and rely solely on the nonlinear nature of the model to provide identification, the coefficient estimates on the up to God and ideal number of children variables are quite stable (i.e., joint and individual Wald tests reveal no significant difference between the up to God and ideal number of children coefficients in the two models at 1% level of significance). This finding is consistent with Mroz’s (1999) Monte Carlo results, which found that the discrete factor model produced relatively stable results even when relying solely on nonlinearities to identify the model.

While the above test is valid, it requires that the model be identified by functional form which some may consider problematic. Therefore, we conduct a second test to provide further evidence that the five identifying variables should be excluded from the family planning equation. Specifically, we re-estimate the model five times, including one and only one of the identifying variables in the family planning equation. Because the model is over-identified, these models are still identified by exclusion restrictions alone. Each of these five models yields a zero coefficient on the lone identifying variable in the family planning equation, again suggesting that these variables are valid exclusion restrictions.

All of the above results suggest that our model is identified and our results are quite robust.

B.3 Endogeneity

The statistically significant random effects discussed in Section 4.4 suggest that there are unobserved factors that affect all three outcome variables. An important question for empiricists is whether or not controlling for this endogeneity actually leads to significantly different point estimates and model predictions.

We first examine whether controlling for endogenous selection into a numeric ideal number of children response has a significant effect on point estimates. Table 6 contains point estimates for the simple model and the preferred model. We find significant and important differences in several coefficients, including those on age, education, and income. Controlling for selection flips the sign on the age coefficients from negative and insignificant to positive and significant. This result is important, as the simple model suggests a potential generational shift towards smaller family sizes, while the model that controls for selection suggests just the opposite. The simple model also suggests that the role education and high income play in reducing a woman’s ideal number of children is slightly overstated when selection is not controlled for.

Next, we examine how the coefficients on the endogenous up to God and ideal number of children variables in the family planning equation are affected by controlling for unobserved heterogeneity (Table 5). In both the simple model and the model with unobserved heterogeneity, unmarried women responding “up to God” and reporting a higher ideal number of children are significantly less likely to use family planning methods than those reporting that they desire only a small number of or no children; however, the point estimates in the two models are different. For married women, these negative relationships are somewhat diminished in the simple model, while the relationships are intensified in the full model.

While comparing the point estimates across these two models gives us a general idea of the role that unobserved heterogeneity is playing, a statistical test is needed to determine whether or not the differences are statistically significant. However, because a logit-type model imposes an error variance of ${\pi^{2}}/3$ and the heterogeneity terms are subsumed into the logistic error in the simpler model, we cannot directly compare point estimates from the two estimation procedures. Thus, we use simulations to test for differences between the simple and preferred model. Specifically, we test whether or not controlling for unobserved heterogeneity in the main model cause the endogenous variables to have different marginal effects on the family planning decision.

Using the procedure described in Section 4.6 of the main text, we first simulate a family planning decision in the simple model, assuming all women leave their family size “up to God”.^[[2]](#footnote-2)^ We then simulate the simple model again, assuming all women respond “zero” to the question of ideal number of children. The model predicts that shifting the population in this way increases the probability of using family planning by 8.5 percentage points (s.e. 0.00063). We then repeat this exercise using the full model, which controls for unobserved heterogeneity. The full model predicts a 12.6 percentage point (s.e. 0.0023) increase in the probability of using family planning. We use a t-test to verify that this difference is significant at a 0.001% level of significance.

We conduct a second set of simulations that are similar to the above, except the population of women is first assumed to have an ideal family size of one child and then zero children. The simple model predicts that this shift would increase the likelihood of family planning usage by 1.13 percentage points (s.e. 0.00017); the full model by 0.98 percentage point (s.e. 0.00022). We use a t-test to verify that this difference is significant at a 0.05% level of significance.

These findings provide clear evidence of the need to control for the endogenous response to the ideal number of children question.

B.4 Traditional Method Users

Among the 145 women who report currently using a traditional family planning method, only 12 report visiting a facility for family planning services. This finding could mean that health facilities are not the primary source of instruction on tradition method use, potentially suggesting traditional and modern methods should be treated differently in our study. That said, community outreach by nearby facilities could impact the use of traditional methods without women ever visiting a facility, particularly if outreach focuses on encouraging women to have fewer children. Ultimately, the extent to which the handling of traditional method users matters can be tested empirically. In Table C below we provided parameter estimates from Equation 3 (i.e., the family planning decision with no unobserved heterogeneity) for two new specification (i) dropping traditional users (column 2) and (ii) treating traditional users as non-users (column 3). Column 1 contains our preferred results; traditional method users are treated as FP users. Notice that there are no significant differences in the parameter estimates under the three different specifications. This finding is not entirely surprising, as a very small proportion of our sample uses these methods (1.5%).

| **Table A: Results with No District Fixed Effects** | | | | | | | | | | | | | | | | | |
| --- | --- | --- | --- | --- | --- | --- | --- | --- | --- | --- | --- | --- | --- | --- | --- | --- | --- |
|  | | Up to God | | |  | | Ideal Kids | | | |  | | Family Planning Use | | | | |
|  | | Coef. |  | SE | |  | | Coef. |  | SE | |  | | Coef. |  | SE |  |
| *Health Facility and Pharmacy Variables* | |  |  |  | |  | |  |  |  | |  | |  |  |  |  |
| Number of public facilities | |  |  |  | |  | |  |  |  | |  | | -0.034 |  | 0.031 |  |
| Number of private facilities | |  |  |  | |  | |  |  |  | |  | | 0.066 |  | 0.041 |  |
| Number of high volume facilities | |  |  |  | |  | |  |  |  | |  | | 0.027 |  | 0.034 |  |
| Number of pharmacies | |  |  |  | |  | |  |  |  | |  | | -0.003 |  | 0.010 |  |
| No facilities/pharmacies participate in survey | |  |  |  | |  | |  |  |  | |  | | -0.292 |  | 0.244 |  |
| Average number of doctors at facility | |  |  |  | |  | |  |  |  | |  | | -0.005 |  | 0.072 |  |
| Average number of nurses at facility | |  |  |  | |  | |  |  |  | |  | | -0.005 |  | 0.024 |  |
| Average number of midwives at facility | |  |  |  | |  | |  |  |  | |  | | -0.016 |  | 0.050 |  |
| Prop. of pharmacies with mult. pharmacists | |  |  |  | |  | |  |  |  | |  | | 0.249 |  | 0.278 |  |
| Prop. of facilities with a family planning protocol | |  |  |  | |  | |  |  |  | |  | | 0.510 | ** | 0.222 |  |
| Prop. of pharmacies requiring FP training | |  |  |  | |  | |  |  |  | |  | | -0.121 |  | 0.159 |  |
| Prop. pharmacies allowing staff to advise on FP | |  |  |  | |  | |  |  |  | |  | | -0.014 |  | 0.178 |  |
| Average FP methods sold at health facility | |  |  |  | |  | |  |  |  | |  | | 0.026 |  | 0.037 |  |
| Average FP methods sold at pharmacy | |  |  |  | |  | |  |  |  | |  | | 0.009 |  | 0.050 |  |
| Any facility has an FP social program | |  |  |  | |  | |  |  |  | |  | | -0.050 |  | 0.099 |  |
| Any pharmacy has a FP social program | |  |  |  | |  | |  |  |  | |  | | 0.266 | ** | 0.107 |  |
| Any facility has a health social worker | | -0.359 | ***** | 0.068 | |  | | -0.118 | *** | 0.044 | |  | | 0.020 |  | 0.095 |  |
| Any facility has IEC outreach program | | -0.076 |  | 0.081 | |  | | 0.080 |  | 0.057 | |  | | -0.105 |  | 0.110 |  |
| Any facility hosts health talks for the comm. | | -0.086 |  | 0.108 | |  | | 0.003 |  | 0.077 | |  | | -0.229 |  | 0.140 |  |
| Prop. giving FP advice during non-FP visit | | 0.832 | *** | 0.149 | |  | | -0.046 |  | 0.101 | |  | | -0.252 |  | 0.205 |  |
| Average number if IEC FP materials at facility | | 0.041 | * | 0.023 | |  | | -0.030 |  | 0.019 | |  | | 0.021 |  | 0.047 |  |
| Number of health facilities with delivery services | | -0.114 | *** | 0.031 | |  | | 0.027 | * | 0.015 | |  | |  |  |  |  |
| *Individual Variables* | |  |  |  | |  | |  |  |  | |  | |  |  |  |  |
| Ideal number of kids | |  |  |  | |  | |  |  |  | |  | | -0.175 | *** | 0.064 |  |
| Number of kids is left "up to God" | |  |  |  | |  | |  |  |  | |  | | -1.191 | *** | 0.384 |  |
| Ideal number of kids * married | |  |  |  | |  | |  |  |  | |  | | 0.121 | * | 0.067 |  |
| Number of kids is left "up to God" * married | |  |  |  | |  | |  |  |  | |  | | 0.512 |  | 0.405 |  |
| Age (reference: 40+) | |  |  |  | |  | |  |  |  | |  | |  |  |  |  |
| 15-19 | | -0.449 | *** | 0.120 | |  | | -0.013 |  | 0.088 | |  | | -2.125 | *** | 0.210 |  |
| 20-24 | | -0.499 | *** | 0.111 | |  | | 0.012 |  | 0.083 | |  | | -0.703 | *** | 0.128 |  |
| 25-29 | | -0.271 | ** | 0.105 | |  | | -0.015 |  | 0.081 | |  | | -0.425 | *** | 0.115 |  |
| 30-34 | | -0.580 | *** | 0.106 | |  | | -0.091 |  | 0.079 | |  | | -0.049 |  | 0.105 |  |
| 35-39 | | -0.455 | *** | 0.106 | |  | | -0.067 |  | 0.081 | |  | | 0.061 |  | 0.104 |  |
| Highest level of education (reference: none) | |  |  |  | |  | |  |  |  | |  | |  |  |  |  |
| Primary school | | -0.359 | *** | 0.067 | |  | | -0.487 | *** | 0.050 | |  | | 0.331 | *** | 0.077 |  |
| Middle school | | -0.601 | *** | 0.110 | |  | | -0.680 | *** | 0.069 | |  | | 0.591 | *** | 0.117 |  |
| High school or higher | | -0.607 | *** | 0.152 | |  | | -0.770 | *** | 0.083 | |  | | 0.411 | *** | 0.143 |  |
| Ethnicity (reference: other) | |  |  |  | |  | |  |  |  | |  | |  |  |  |  |
| Wolof | | 0.150 | * | 0.086 | |  | | 0.029 |  | 0.055 | |  | | -0.093 |  | 0.088 |  |
| Poular | | 0.125 |  | 0.095 | |  | | -0.086 |  | 0.061 | |  | | -0.071 |  | 0.099 |  |
| Serer | | -0.036 |  | 0.098 | |  | | 0.117 | * | 0.061 | |  | | -0.143 |  | 0.100 |  |
| Socioeconomic status (reference: 1st quintile) | |  |  |  | |  | |  |  |  | |  | |  |  |  |  |
| 2nd quintile | | 0.123 |  | 0.089 | |  | | -0.112 | * | 0.064 | |  | | 0.071 |  | 0.101 |  |
| 3rd quintile | | 0.061 |  | 0.095 | |  | | -0.138 | ** | 0.066 | |  | | 0.059 |  | 0.102 |  |
| 4th quintile | | -0.080 |  | 0.107 | |  | | -0.214 | *** | 0.072 | |  | | 0.011 |  | 0.105 |  |
| 5th quintile | | -0.061 |  | 0.124 | |  | | -0.214 | *** | 0.082 | |  | | -0.126 |  | 0.111 |  |
| Muslim | | 0.673 | *** | 0.188 | |  | | 0.489 | *** | 0.085 | |  | | -0.345 | ** | 0.141 |  |
| Worked last week | | 0.021 |  | 0.060 | |  | | 0.034 |  | 0.042 | |  | | 0.239 | *** | 0.064 |  |
| Listens to radio | | -0.387 | *** | 0.062 | |  | | -0.029 |  | 0.045 | |  | | 0.007 |  | 0.071 |  |
| Reads the newspaper/magazines | | -0.238 | *** | 0.091 | |  | | -0.118 | ** | 0.053 | |  | | -0.050 |  | 0.090 |  |
| Has a cell phone | | 0.096 |  | 0.064 | |  | | 0.012 |  | 0.045 | |  | | 0.004 |  | 0.077 |  |
| Has internet access | | -0.721 | *** | 0.145 | |  | | -0.135 | ** | 0.063 | |  | | -0.118 |  | 0.125 |  |
| Married | | -0.430 | * | 0.249 | |  | | 0.309 | * | 0.183 | |  | | 1.638 | *** | 0.387 |  |
| Partner: other wives | | 0.146 | * | 0.079 | |  | | -0.026 |  | 0.063 | |  | | -0.188 | ** | 0.080 |  |
| Partner: age | | 0.132 | *** | 0.046 | |  | | 0.055 |  | 0.035 | |  | | -0.119 | ** | 0.048 |  |
| Partner: age is not known | | 0.872 | *** | 0.215 | |  | | 0.181 |  | 0.161 | |  | | -0.806 | *** | 0.228 |  |
| Partner: highest education (reference: none) | |  |  |  | |  | |  |  |  | |  | |  |  |  |  |
| Primary school | | -0.299 | ** | 0.129 | |  | | -0.105 |  | 0.089 | |  | | 0.478 | *** | 0.113 |  |
| Middle school | | -0.814 | *** | 0.177 | |  | | -0.184 | * | 0.099 | |  | | 0.154 |  | 0.131 |  |
| High school or higher | | -0.115 |  | 0.127 | |  | | -0.149 | * | 0.086 | |  | | 0.266 | ** | 0.114 |  |
| Educated, but unsure of grade | | -0.090 |  | 0.083 | |  | | -0.279 | *** | 0.067 | |  | | 0.240 | *** | 0.087 |  |
| Partner: works | | 0.195 |  | 0.119 | |  | | -0.076 |  | 0.092 | |  | | 0.079 |  | 0.117 |  |
| Number of beds in the home | | 0.015 |  | 0.012 | |  | | 0.041 | *** | 0.008 | |  | |  |  |  |  |
| Employ help in the home | | -0.029 |  | 0.088 | |  | | -0.093 | * | 0.054 | |  | |  |  |  |  |
| Running water in the home | | -0.118 | * | 0.064 | |  | | 0.011 |  | 0.042 | |  | |  |  |  |  |
| Toilet in the home | | 0.335 | *** | 0.094 | |  | | -0.145 | ** | 0.059 | |  | |  |  |  |  |
| Constant | | -1.975 | *** | 0.252 | |  | | 4.722 | *** | 0.147 | |  | | -1.692 | *** | 0.373 |  |
| District Fixed Effects | | No | | |  | | No | | | |  | | No | | | | |
| Observations | | 9272 | | |  | | 7519 | | | |  | | 9272 | | | | |

* statistically significant at the 10% level.

** statistically significant at the 5% level.

*** statistically significant at the 1% level.

| **Table B: Family Planning Use, Including Exclusion Restrictions** | | | |
| --- | --- | --- | --- |
|  | Coef |  | SE |
| *Health Facility and Pharmacy Variables* |  |  |  |
| **Number of health facilities with delivery services** | -0.013 |  | 0.105 |
| Number of public facilities | 0.006 |  | 0.094 |
| Number of private facilities | -0.058 |  | 0.115 |
| Number of high volume facilities | 0.034 |  | 0.117 |
| Number of pharmacies | -0.030 |  | 0.028 |
| No facilities/pharmacies participate in survey | -0.306 |  | 0.547 |
| Average number of doctors at facility | -0.130 |  | 0.226 |
| Average number of nurses at facility | 0.084 |  | 0.076 |
| Average number of midwives at facility | -0.076 |  | 0.126 |
| Prop. of pharmacies with mult. pharmacists | 2.005 | *** | 0.776 |
| Prop. of facilities with a family planning protocol | 0.781 | * | 0.465 |
| Prop. of pharmacies requiring FP training | -0.533 |  | 0.461 |
| Prop. of pharmacies allowing staff to advise on FP | -0.847 | * | 0.474 |
| Average FP methods sold at health facility | -0.032 |  | 0.095 |
| Average FP methods sold at pharmacy | 0.190 |  | 0.132 |
| Any facility has an FP social program | 0.225 |  | 0.259 |
| Any pharmacy has a FP social program | 0.391 |  | 0.293 |
| Any facility has a health social worker | 0.153 |  | 0.267 |
| Any facility has IEC outreach program | -0.175 |  | 0.238 |
| Any facility hosts health talks for the comm. | -0.555 |  | 0.387 |
| Prop. giving FP advice during non-FP visit | 0.365 |  | 0.527 |
| Average number if IEC FP materials at facility | 0.020 |  | 0.117 |
| *Individual Variables* |  |  |  |
| **Number of beds in the home** | -0.008 |  | 0.024 |
| **Employ help in the home** | 0.008 |  | 0.164 |
| **Running water in the home** | -0.082 |  | 0.131 |
| **Toilet in the home** | 0.101 |  | 0.200 |
| Ideal number of kids | -0.207 | ** | 0.090 |
| Number of kids is left "up to God" | -1.605 | *** | 0.592 |
| Ideal number of kids * married | -0.128 |  | 0.120 |
| Number of kids is left "up to God" * married | -1.616 | * | 0.893 |
| Age (reference: 40+) |  |  |  |
| 15-19 | -2.943 | *** | 0.354 |
| 20-24 | -1.244 | *** | 0.254 |
| 25-29 | -0.770 | *** | 0.224 |
| 30-34 | -0.122 |  | 0.212 |
| 35-39 | 0.127 |  | 0.212 |
| Highest level of education (reference: none) |  |  |  |
| Primary school | 0.504 | *** | 0.160 |
| Middle school | 1.024 | *** | 0.234 |
| High school or higher | 0.445 |  | 0.286 |
| Ethnicity (reference: other) |  |  |  |
| Wolof | -0.281 |  | 0.191 |
| Poular | -0.279 |  | 0.210 |
| Serer | -0.357 | * | 0.207 |
| Socioeconomic status (reference: 1^st^ quintile) |  |  |  |
| 2nd quintile | 0.041 |  | 0.192 |
| 3rd quintile | 0.110 |  | 0.197 |
| 4th quintile | -0.050 |  | 0.219 |
| 5th quintile | -0.332 |  | 0.248 |
| Muslim | -0.922 | *** | 0.296 |
| Worked last week | 0.365 | *** | 0.122 |
| Listens to radio | 0.011 |  | 0.130 |
| Reads the newspaper/magazines | -0.216 |  | 0.177 |
| Has a cell phone | 0.119 |  | 0.138 |
| Has internet access | -0.108 |  | 0.230 |
| Married | 3.961 | *** | 0.824 |
| Partner: other wives | -0.203 |  | 0.181 |
| Partner: age | -0.157 | * | 0.092 |
| Partner: age is not known | -1.091 | ** | 0.441 |
| Partner: highest education (reference: none) |  |  |  |
| Primary school | 1.116 | *** | 0.362 |
| Middle school | 0.394 |  | 0.354 |
| High school or higher | 0.656 | ** | 0.267 |
| Educated, but unsure of grade | 0.481 | *** | 0.179 |
| Partner: works | 0.194 |  | 0.249 |
| Constant | -13.351 | *** | 1.965 |
| *DFRE Variables* |  |  |  |
| Point 1 (Normalized to Zero) | 0.000 |  | 0.000 |
| Point 2 | 12.500 | *** | 2.010 |
| Point 3 | 13.143 | *** | 1.935 |
| District Fixed Effects | Yes | | |
| Observations | 9263 | | |

* statistically significant at the 10% level.

** statistically significant at the 5% level.

*** statistically significant at the 1% level.

| **Table C: Results for Any Use of Family Planning Methods; Different Handling of Traditional Method Users** | | | | | | | | | | | |
| --- | --- | --- | --- | --- | --- | --- | --- | --- | --- | --- | --- |
|  | Full Sample | | |  | Drop Traditional FP Users | | |  | Recode Traditional FP Users | | |
|  | Coef. |  | SE |  | Coef. |  | SE |  | Coef. |  | SE |
| *Individual Variables* |  |  |  |  |  |  |  |  |  |  |  |
| Constant | -2.272 | *** | 0.637 |  | -2.357 | *** | 0.652 |  | -2.411 | *** | 0.650 |
| Ideal number of kids | -0.178 | *** | 0.065 |  | -0.186 | *** | 0.066 |  | -0.191 | *** | 0.066 |
| Number of kids is left "up to God" | -1.155 | *** | 0.388 |  | -1.248 | *** | 0.398 |  | -1.286 | *** | 0.398 |
| Ideal number of kids * married | 0.130 | * | 0.068 |  | 0.127 | * | 0.070 |  | 0.130 | * | 0.070 |
| Number of kids is left "up to God" * married | 0.518 |  | 0.410 |  | 0.579 |  | 0.421 |  | 0.626 |  | 0.420 |
| Age (reference 40+) |  |  |  |  |  |  |  |  |  |  |  |
| 15-19 | -2.120 | *** | 0.211 |  | -2.120 | *** | 0.220 |  | -2.087 | *** | 0.219 |
| 20-24 | -0.695 | *** | 0.129 |  | -0.663 | *** | 0.133 |  | -0.628 | *** | 0.133 |
| 25-29 | -0.427 | *** | 0.116 |  | -0.401 | *** | 0.120 |  | -0.370 | *** | 0.119 |
| 30-34 | -0.043 |  | 0.106 |  | 0.006 |  | 0.110 |  | 0.028 |  | 0.109 |
| 35-39 | 0.054 |  | 0.105 |  | 0.064 |  | 0.109 |  | 0.073 |  | 0.108 |
| Highest level of education (reference: none) |  |  |  |  |  |  |  |  |  |  |  |
| Primary school | 0.339 | *** | 0.078 |  | 0.311 | *** | 0.080 |  | 0.296 | *** | 0.080 |
| Middle school | 0.613 | *** | 0.118 |  | 0.558 | *** | 0.122 |  | 0.530 | *** | 0.121 |
| High school or higher | 0.433 | *** | 0.145 |  | 0.310 | ** | 0.151 |  | 0.267 | * | 0.150 |
| Ethnicity (reference: other) |  |  |  |  |  |  |  |  |  |  |  |
| Wolof | -0.076 |  | 0.090 |  | -0.066 |  | 0.093 |  | -0.060 |  | 0.092 |
| Poular | -0.036 |  | 0.100 |  | -0.062 |  | 0.104 |  | -0.069 |  | 0.104 |
| Serer | -0.139 |  | 0.101 |  | -0.110 |  | 0.104 |  | -0.087 |  | 0.104 |
| Socioeconomic Status (reference: 1st quintile) |  |  |  |  |  |  |  |  |  |  |  |
| 2nd quintile | 0.078 |  | 0.103 |  | 0.065 |  | 0.106 |  | 0.065 |  | 0.106 |
| 3rd quintile | 0.067 |  | 0.103 |  | 0.071 |  | 0.107 |  | 0.067 |  | 0.106 |
| 4th quintile | 0.006 |  | 0.107 |  | 0.037 |  | 0.111 |  | 0.045 |  | 0.110 |
| 5th quintile | -0.138 |  | 0.114 |  | -0.088 |  | 0.117 |  | -0.060 |  | 0.116 |
| Muslim | -0.324 | ** | 0.145 |  | -0.315 | ** | 0.150 |  | -0.286 | * | 0.149 |
| Worked last week | 0.241 | *** | 0.065 |  | 0.253 | *** | 0.067 |  | 0.246 | *** | 0.067 |
| listens to radio | 0.002 |  | 0.072 |  | -0.004 |  | 0.074 |  | -0.009 |  | 0.074 |
| Reads the newspaper/magazines | -0.074 |  | 0.091 |  | -0.094 |  | 0.094 |  | -0.115 |  | 0.094 |
| Has a cell phone | 0.005 |  | 0.078 |  | -0.013 |  | 0.080 |  | -0.015 |  | 0.079 |
| Has internet access | -0.144 |  | 0.128 |  | -0.163 |  | 0.134 |  | -0.168 |  | 0.133 |
| Married | 1.660 | *** | 0.393 |  | 1.499 | *** | 0.404 |  | 1.422 | *** | 0.403 |
| Partner: has other wives | -0.195 | ** | 0.080 |  | -0.183 | ** | 0.083 |  | -0.164 | ** | 0.082 |
| Partner: age | -0.120 | ** | 0.049 |  | -0.114 | ** | 0.050 |  | -0.113 | ** | 0.050 |
| Partner: age is not known | -0.800 | *** | 0.230 |  | -0.749 | *** | 0.237 |  | -0.729 | *** | 0.236 |
| Partner: highest education (reference: none) |  |  |  |  |  |  |  |  |  |  |  |
| Primary school | 0.475 | *** | 0.114 |  | 0.515 | *** | 0.117 |  | 0.507 | *** | 0.117 |
| Middle school | 0.155 |  | 0.133 |  | 0.202 |  | 0.137 |  | 0.209 |  | 0.136 |
| High school or higher | 0.281 | ** | 0.115 |  | 0.262 | ** | 0.120 |  | 0.238 | ** | 0.119 |
| Educated, but usure of grade | 0.253 | *** | 0.088 |  | 0.251 | *** | 0.091 |  | 0.240 | *** | 0.090 |
| Partner works | 0.050 |  | 0.119 |  | 0.103 |  | 0.125 |  | 0.104 |  | 0.124 |
| *Health Facility and Pharmacy Variables* |  |  |  |  |  |  |  |  |  |  |  |
| Number of public facilities | 0.015 |  | 0.047 |  | 0.035 |  | 0.049 |  | 0.044 |  | 0.048 |
| Number of private facilities | -0.015 |  | 0.060 |  | -0.014 |  | 0.062 |  | -0.006 |  | 0.062 |
| Number of pharmacies | -0.009 |  | 0.062 |  | -0.011 |  | 0.064 |  | -0.012 |  | 0.064 |
| Number of high volume facilities | -0.008 |  | 0.015 |  | -0.008 |  | 0.015 |  | -0.007 |  | 0.015 |
| No facilities/pharmacies participate in survey | -0.218 |  | 0.331 |  | -0.250 |  | 0.348 |  | -0.239 |  | 0.347 |
| Average number of doctors at facility | -0.095 |  | 0.121 |  | -0.112 |  | 0.124 |  | -0.135 |  | 0.123 |
| Average number of nurses at facility | 0.021 |  | 0.040 |  | 0.032 |  | 0.041 |  | 0.040 |  | 0.041 |
| Average number of midwives at facility | 0.006 |  | 0.070 |  | 0.003 |  | 0.072 |  | 0.010 |  | 0.072 |
| Prop. of pharmacies with mult. pharmacisits | 0.889 | ** | 0.433 |  | 0.843 | * | 0.450 |  | 0.829 | * | 0.447 |
| Prop. of facilities with a family planning protocol | 0.285 |  | 0.257 |  | 0.250 |  | 0.266 |  | 0.234 |  | 0.264 |
| Prop. of pharmacies requiring FP training | -0.215 |  | 0.247 |  | -0.254 |  | 0.253 |  | -0.266 |  | 0.253 |
| Prop. Of pharmacies allowing staff to advise on FP | -0.176 |  | 0.241 |  | -0.279 |  | 0.250 |  | -0.329 |  | 0.248 |
| Average FP methods sold at health facility | -0.012 |  | 0.052 |  | -0.013 |  | 0.054 |  | -0.012 |  | 0.053 |
| Average FP methods sold at pharmacy | 0.059 |  | 0.067 |  | 0.080 |  | 0.069 |  | 0.088 |  | 0.069 |
| Any facility has FP social program | -0.051 |  | 0.131 |  | -0.060 |  | 0.135 |  | -0.055 |  | 0.134 |
| Any pharmacy has FP social program | 0.115 |  | 0.163 |  | 0.083 |  | 0.167 |  | 0.076 |  | 0.166 |
| Any facility has a health social worker | 0.179 |  | 0.147 |  | 0.193 |  | 0.150 |  | 0.201 |  | 0.150 |
| Some facility has comm. outreach program | -0.123 |  | 0.129 |  | -0.175 |  | 0.132 |  | -0.199 |  | 0.131 |
| Some facility conducts comm. Talks on FP | -0.100 |  | 0.202 |  | -0.062 |  | 0.209 |  | -0.031 |  | 0.208 |
| Prop. giving FP advice during non-FP visit | -0.041 |  | 0.294 |  | 0.049 |  | 0.303 |  | 0.066 |  | 0.302 |
| Average number if IEC materials at facility | 0.041 |  | 0.063 |  | 0.051 |  | 0.064 |  | 0.052 |  | 0.064 |
| District Fixed Effects | Yes | | |  | Yes | | |  | Yes | | |
| Observations | 9263 | | |  | 9128 | | |  | 9263 | | |
| *Notes:* This table contains parameter estimates from a logit regression of any FP use on controls under three different specifications. Our preferred specification can be found in Column 1 (these results are identical to those in Table 5, Column 1); here, traditional FP users are categorized as FP users. In Column 2, traditional FP users are dropped. In Column 3, traditional FP users are treated as non-users.  * statistically significant at the 10% level.  ** statistically significant at the 5% level.  *** statistically significant at the 1% level. | | | | | | | | | | | |
|  |  |  |  |  |  |  |  |  |  |  |  |
|  |  |  |  |  |  |  |  |  |  |  |  |
|  |  |  |  |  |  |  |  |  |  |  |  |

| **Table D: Individual-Level Independent Variables, Non-Responders** | | | | | |
| --- | --- | --- | --- | --- | --- |
|  | Obs. |  | Mean |  | SD |
| Age | 351 |  | 31.17 |  | 10.03 |
| Education |  |  |  |  |  |
| None | 351 |  | 0.47 |  |  |
| Primary school | 351 |  | 0.33 |  |  |
| Middle school | 351 |  | 0.15 |  |  |
| High school or higher | 351 |  | 0.05 |  |  |
| Income | 351 |  | 3.58 |  | 2.71 |
| Muslim | 351 |  | 0.93 |  |  |
| Ethnicity |  |  |  |  |  |
| Wolof | 351 |  | 0.23 |  |  |
| Poular | 351 |  | 0.18 |  |  |
| Serer | 351 |  | 0.20 |  |  |
| Other | 351 |  | 0.39 |  |  |
| Worked in last week | 339 |  | 0.45 |  |  |
| Listens to radio | 340 |  | 0.67 |  |  |
| Reads newspaper/magazine | 338 |  | 0.19 |  |  |
| Has personal cell phone | 337 |  | 0.70 |  |  |
| Has internet access | 337 |  | 0.07 |  |  |
| Number of beds in the home | 351 |  | 4.66 |  | 3.10 |
| Employs help in the home | 350 |  | 0.17 |  |  |
| Running water in the home | 351 |  | 0.27 |  |  |
| Toilet in the home | 351 |  | 0.81 |  |  |
| Married* | 342 |  | 0.66 |  |  |
| Partner: other wives | 222 |  | 0.29 |  |  |
| Partner: age | 225 |  | 35.60 |  | 21.77 |
| Partner: education |  |  |  |  |  |
| None | 223 |  | 0.41 |  |  |
| Primary school | 223 |  | 0.10 |  |  |
| Middle school | 223 |  | 0.08 |  |  |
| High school or higher | 223 |  | 0.11 |  |  |
| Educated, but unsure of grade | 223 |  | 0.30 |  |  |
| Partner: works | 220 |  | 0.85 |  |  |
| * Mean and standard deviation for all variables below 'married' are calculated for married individuals only. | | | | | |
|  |  |  |  |  |  |
|  |  |  |  |  |  |

| **Table E1: Results with Health Service Area Defined as 0.5 KM Radius Around PSU Centroid** | | | | | | | | | | | |
| --- | --- | --- | --- | --- | --- | --- | --- | --- | --- | --- | --- |
|  | Up to God | | |  | Ideal Kids | | |  | Family Planning Use | | |
|  | Coef. |  | SE |  | Coef. |  | SE |  | Coef. |  | SE |
| *Health Facility and Pharmacy Variables* |  |  |  |  |  |  |  |  |  |  |  |
| Number of public facilities |  |  |  |  |  |  |  |  | -0.111 |  | 0.151 |
| Number of private facilities |  |  |  |  |  |  |  |  | -0.177 |  | 0.164 |
| Number of high volume facilities |  |  |  |  |  |  |  |  | -0.011 |  | 0.198 |
| Number of pharmacies |  |  |  |  |  |  |  |  | 0.120 | ** | 0.057 |
| No facilities/pharmacies participate in survey |  |  |  |  |  |  |  |  | 0.176 |  | 0.582 |
| Average number of doctors at facility |  |  |  |  |  |  |  |  | -0.089 |  | 0.092 |
| Average number of nurses at facility |  |  |  |  |  |  |  |  | 0.010 |  | 0.032 |
| Average number of midwives at facility |  |  |  |  |  |  |  |  | 0.085 |  | 0.065 |
| Prop. of pharmacies with mult. pharmacists |  |  |  |  |  |  |  |  | 0.182 |  | 0.355 |
| Prop. of facilities with a family planning protocol |  |  |  |  |  |  |  |  | 0.051 |  | 0.290 |
| Prop. of pharmacies requiring FP training |  |  |  |  |  |  |  |  | -0.144 |  | 0.261 |
| Prop. pharmacies allowing staff to advise on FP |  |  |  |  |  |  |  |  | 0.381 |  | 0.280 |
| Average FP methods sold at health facility |  |  |  |  |  |  |  |  | 0.120 | * | 0.064 |
| Average FP methods sold at pharmacy |  |  |  |  |  |  |  |  | -0.033 |  | 0.077 |
| Any facility has an FP social program |  |  |  |  |  |  |  |  | -0.906 | ** | 0.449 |
| Any pharmacy has a FP social program |  |  |  |  |  |  |  |  | -0.198 |  | 0.395 |
| Any facility has a health social worker |  |  |  |  |  |  |  |  | 0.278 |  | 0.300 |
| Any facility has IEC outreach program |  |  |  |  |  |  |  |  | 0.041 |  | 0.241 |
| Any facility hosts health talks for the comm. |  |  |  |  |  |  |  |  | -0.124 |  | 0.246 |
| Prop. giving FP advice during non-FP visit |  |  |  |  |  |  |  |  | -0.340 |  | 0.343 |
| Average number if IEC FP materials at facility |  |  |  |  |  |  |  |  | -0.036 |  | 0.074 |
| Number of health facilities with delivery services | 0.351 | *** | 0.076 |  | 0.002 |  | 0.037 |  |  |  |  |
| *Individual Variables* |  |  |  |  |  |  |  |  |  |  |  |
| Ideal number of kids |  |  |  |  |  |  |  |  | -0.169 | * | 0.097 |
| Number of kids is left "up to God" |  |  |  |  |  |  |  |  | 0.905 |  | 0.904 |
| Ideal number of kids * married |  |  |  |  |  |  |  |  | -0.020 |  | 0.111 |
| Number of kids is left "up to God" * married |  |  |  |  |  |  |  |  | 0.315 |  | 0.835 |
| Age (reference: 40+) |  |  |  |  |  |  |  |  |  |  |  |
| 15-19 | -0.520 | *** | 0.139 |  | 0.143 | * | 0.077 |  | -2.837 | *** | 0.351 |
| 20-24 | -0.569 | *** | 0.131 |  | 0.185 | *** | 0.074 |  | -1.070 | *** | 0.250 |
| 25-29 | -0.331 | *** | 0.122 |  | 0.157 | ** | 0.072 |  | -0.603 | *** | 0.217 |
| 30-34 | -0.681 | *** | 0.125 |  | 0.071 |  | 0.071 |  | 0.220 |  | 0.205 |
| 35-39 | -0.537 | *** | 0.125 |  | 0.068 |  | 0.074 |  | 0.350 |  | 0.219 |
| Highest level of education (reference: none) |  |  |  |  |  |  |  |  |  |  |  |
| Primary school | -0.396 | *** | 0.078 |  | -0.316 | *** | 0.043 |  | 0.671 | *** | 0.149 |
| Middle school | -0.707 | *** | 0.127 |  | -0.431 | *** | 0.057 |  | 1.318 | *** | 0.256 |
| High school or higher | -0.737 | *** | 0.168 |  | -0.523 | *** | 0.065 |  | 0.837 | *** | 0.256 |
| Ethnicity (reference: other) |  |  |  |  |  |  |  |  |  |  |  |
| Wolof | 0.159 | * | 0.097 |  | 0.034 |  | 0.045 |  | -0.225 |  | 0.173 |
| Poular | 0.136 |  | 0.107 |  | -0.071 |  | 0.050 |  | -0.213 |  | 0.190 |
| Serer | -0.007 |  | 0.110 |  | 0.041 |  | 0.049 |  | -0.244 |  | 0.189 |
| Socioeconomic status (reference: 1st quintile) |  |  |  |  |  |  |  |  |  |  |  |
| 2nd quintile | 0.083 |  | 0.102 |  | -0.053 |  | 0.055 |  | 0.024 |  | 0.189 |
| 3rd quintile | -0.013 |  | 0.107 |  | -0.092 | * | 0.055 |  | 0.001 |  | 0.192 |
| 4th quintile | -0.173 |  | 0.119 |  | -0.142 | ** | 0.059 |  | -0.098 |  | 0.201 |
| 5th quintile | -0.080 |  | 0.141 |  | -0.140 | ** | 0.067 |  | -0.330 |  | 0.209 |
| Muslim | 0.693 | *** | 0.202 |  | 0.230 |  | 0.167 |  | -0.842 | *** | 0.330 |
| Worked last week | -0.007 |  | 0.069 |  | 0.031 |  | 0.035 |  | 0.454 | *** | 0.127 |
| Listens to radio | -0.449 | *** | 0.071 |  | 0.051 |  | 0.038 |  | 0.062 |  | 0.129 |
| Reads the newspaper/magazines | -0.303 | *** | 0.102 |  | -0.093 | ** | 0.042 |  | -0.134 |  | 0.182 |
| Has a cell phone | 0.123 | * | 0.073 |  | 0.021 |  | 0.039 |  | 0.018 |  | 0.136 |
| Has internet access | -0.773 | *** | 0.151 |  | -0.160 | *** | 0.047 |  | -0.041 |  | 0.210 |
| Married | -0.500 | * | 0.283 |  | 0.230 |  | 0.167 |  | 3.699 | *** | 0.817 |
| Partner: other wives | 0.179 | * | 0.092 |  | -0.082 |  | 0.056 |  | -0.394 | ** | 0.172 |
| Partner: age | 0.162 | *** | 0.053 |  | 0.055 | * | 0.033 |  | -0.245 | ** | 0.098 |
| Partner: age is not known | 0.997 | *** | 0.249 |  | 0.243 | * | 0.146 |  | -1.749 | *** | 0.490 |
| Partner: highest education (reference: none) |  |  |  |  |  |  |  |  |  |  |  |
| Primary school | -0.309 | *** | 0.145 |  | -0.099 |  | 0.076 |  | 1.176 | *** | 0.300 |
| Middle school | -0.988 | *** | 0.197 |  | -0.066 |  | 0.079 |  | 0.722 | ** | 0.317 |
| High school or higher | -0.139 |  | 0.143 |  | -0.123 | * | 0.073 |  | 0.641 | *** | 0.239 |
| Educated, but unsure of grade | -0.129 |  | 0.097 |  | -0.222 | *** | 0.059 |  | 0.439 | ** | 0.178 |
| Partner: works | 0.215 |  | 0.139 |  | -0.049 |  | 0.083 |  | 0.174 |  | 0.249 |
| Number of beds in the home | 0.021 |  | 0.014 |  | 0.031 | *** | 0.007 |  |  |  |  |
| Employ help in the home | -0.144 |  | 0.099 |  | -0.045 |  | 0.043 |  |  |  |  |
| Running water in the home | -0.027 |  | 0.074 |  | 0.038 |  | 0.035 |  |  |  |  |
| Toilet in the home | 0.428 | *** | 0.107 |  | -0.140 | *** | 0.050 |  |  |  |  |
| Constant | -1.051 | *** | 0.293 |  | 4.226 | *** | 0.131 |  | -6.026 | *** | 0.972 |
| *DFRE Variables* |  |  |  |  |  |  |  |  |  |  |  |
| Point 1 (Normalized to Zero) | 0.000 |  | 0.000 |  | 0.000 |  | 0.000 |  | 0.000 |  | 0.000 |
| Point 2 | -29.428 | *** | 0.000 |  | 4.782 | *** | 0.130 |  | 4.007 | *** | 1.077 |
| Point 3 | -1.908 | *** | 0.512 |  | 0.023 |  | 0.098 |  | 5.190 | *** | 0.945 |
| District Fixed Effects | Yes | | |  | Yes | | |  | Yes | | |
| Joint Log-Likelihood Function Value | -20687.6 | | | | | | | | | | |
| Observations | 9263 | | |  | 7519 | | |  | 9263 | | |
| *Notes*: An upward testing approach (described in Section 4.4 of the paper) is used to determine the optimal number of mass points. The log-likelihood function value at the maximum for the model with HSEs defined by a 1 KM radius around a PSU centroid is -20677.3. The estimated share of individuals represented by each mass point in the 0.5 KM model is 63%, 4%, 33%, respectively. | | | | | | | | | | | |
|  |  |  |  |  |  |  |  |  |  |  |  |
|  |  |  |  |  |  |  |  |  |  |  |  |
| * statistically significant at the 10% level. |  |  |  |  |  |  |  |  |  |  |  |
| ** statistically significant at the 5% level. |  |  |  |  |  |  |  |  |  |  |  |
| *** statistically significant at the 1% level. |  |  |  |  |  |  |  |  |  |  |  |

| **Table E2: Results with Health Service Area Defined as 2 KM Radius Around PSU Centroid** | | | | | | | | | | | |
| --- | --- | --- | --- | --- | --- | --- | --- | --- | --- | --- | --- |
|  | Up to God | | |  | Ideal Kids | | |  | Family Planning Use | | |
|  | Coef. |  | SE |  | Coef. |  | SE |  | Coef. |  | SE |
| *Health Facility and Pharmacy Variables* |  |  |  |  |  |  |  |  |  |  |  |
| Number of public facilities |  |  |  |  |  |  |  |  | -0.161 | ** | 0.068 |
| Number of private facilities |  |  |  |  |  |  |  |  | 0.161 | * | 0.083 |
| Number of high volume facilities |  |  |  |  |  |  |  |  | 0.147 | * | 0.083 |
| Number of pharmacies |  |  |  |  |  |  |  |  | 0.001 |  | 0.018 |
| No facilities/pharmacies participate in survey |  |  |  |  |  |  |  |  | 0.000 |  | 0.000 |
| Average number of doctors at facility |  |  |  |  |  |  |  |  | 0.193 |  | 0.251 |
| Average number of nurses at facility |  |  |  |  |  |  |  |  | 0.043 |  | 0.122 |
| Average number of midwives at facility |  |  |  |  |  |  |  |  | -0.352 |  | 0.269 |
| Prop. of pharmacies with mult. pharmacisits |  |  |  |  |  |  |  |  | 1.250 |  | 1.789 |
| Prop. of facilities with a family planning protocol |  |  |  |  |  |  |  |  | 1.546 |  | 0.941 |
| Prop. of pharmacies requiring FP training |  |  |  |  |  |  |  |  | -0.251 |  | 1.243 |
| Prop. pharmacies allowing staff to advise on FP |  |  |  |  |  |  |  |  | -1.187 |  | 1.156 |
| Average FP methods sold at health facility |  |  |  |  |  |  |  |  | -0.069 |  | 0.166 |
| Average FP methods sold at pharmacy |  |  |  |  |  |  |  |  | 0.314 |  | 0.299 |
| Any facility has an FP social program |  |  |  |  |  |  |  |  | -0.015 |  | 0.214 |
| Any pharmacy has a FP social program |  |  |  |  |  |  |  |  | -0.532 | ** | 0.259 |
| Any facility has a health social worker | -0.619 | *** | 0.224 |  | -0.198 | *** | 0.055 |  | 0.186 |  | 0.296 |
| Any facility has IEC outreach program | 0.795 |  | 0.766 |  | -0.511 | ** | 0.203 |  | 0.331 |  | 0.934 |
| Any facility hosts health talks for the comm. | 1.116 |  | 0.905 |  | 0.555 | *** | 0.195 |  | 0.965 |  | 0.655 |
| Prop. giving FP advice during non-FP visit | 0.000 |  | 0.782 |  | -0.401 |  | 0.278 |  | 0.251 |  | 1.433 |
| Average number if IEC FP materials at facility | 0.003 |  | 0.118 |  | 0.069 | * | 0.043 |  | 0.344 |  | 0.277 |
| Number of health facilities with delivery services | -0.010 |  | 0.031 |  | 0.007 |  | 0.010 |  |  |  |  |
| *Individual Variables* |  |  |  |  |  |  |  |  |  |  |  |
| Ideal number of kids |  |  |  |  |  |  |  |  | -0.242 | *** | 0.090 |
| Number of kids is left "up to God" |  |  |  |  |  |  |  |  | -1.485 |  | 1.019 |
| Ideal number of kids * married |  |  |  |  |  |  |  |  | -0.135 |  | 0.118 |
| Number of kids is left "up to God" * married |  |  |  |  |  |  |  |  | -1.799 | ** | 0.878 |
| Age (reference: 40+) |  |  |  |  |  |  |  |  |  |  |  |
| 15-19 | -0.763 | *** | 0.226 |  | 0.139 | * | 0.077 |  | -2.884 | *** | 0.341 |
| 20-24 | -0.781 | *** | 0.194 |  | 0.180 | ** | 0.074 |  | -1.158 | *** | 0.237 |
| 25-29 | -0.429 | ** | 0.172 |  | 0.159 | ** | 0.071 |  | -0.680 | *** | 0.201 |
| 30-34 | -0.917 | *** | 0.221 |  | 0.071 |  | 0.071 |  | -0.081 |  | 0.218 |
| 35-39 | -0.752 | *** | 0.189 |  | 0.071 |  | 0.074 |  | 0.215 |  | 0.210 |
| Highest level of education (reference: none) |  |  |  |  |  |  |  |  |  |  |  |
| Primary school | -0.559 | *** | 0.149 |  | -0.306 | *** | 0.044 |  | 0.471 | *** | 0.165 |
| Middle school | -1.075 | *** | 0.323 |  | -0.416 | *** | 0.056 |  | 0.999 | *** | 0.263 |
| High school or higher | -1.091 | *** | 0.322 |  | -0.509 | *** | 0.065 |  | 0.418 |  | 0.300 |
| Ethnicity (reference: other) |  |  |  |  |  |  |  |  |  |  |  |
| Wolof | 0.232 |  | 0.150 |  | 0.013 |  | 0.045 |  | -0.267 |  | 0.174 |
| Poular | 0.180 |  | 0.155 |  | -0.081 | * | 0.050 |  | -0.251 |  | 0.190 |
| Serer | -0.029 |  | 0.160 |  | 0.033 |  | 0.049 |  | -0.337 | * | 0.188 |
| Socioeconomic status (reference: 1st quintile) |  |  |  |  |  |  |  |  |  |  |  |
| 2nd quintile | 0.118 |  | 0.142 |  | -0.038 |  | 0.055 |  | 0.053 |  | 0.183 |
| 3rd quintile | 0.031 |  | 0.153 |  | -0.068 |  | 0.055 |  | 0.098 |  | 0.180 |
| 4th quintile | -0.256 |  | 0.184 |  | -0.115 | * | 0.059 |  | -0.061 |  | 0.182 |
| 5th quintile | -0.106 |  | 0.204 |  | -0.107 | * | 0.067 |  | -0.351 | * | 0.193 |
| Muslim | 0.922 | *** | 0.331 |  | 0.342 | *** | 0.061 |  | -0.841 | *** | 0.289 |
| Worked last week | -0.028 |  | 0.099 |  | 0.024 |  | 0.035 |  | 0.349 | *** | 0.125 |
| Listens to radio | -0.653 | *** | 0.141 |  | 0.041 |  | 0.038 |  | 0.024 |  | 0.130 |
| Reads the newspaper/magazines | -0.511 | ** | 0.214 |  | -0.089 | *** | 0.042 |  | -0.184 |  | 0.167 |
| Has a cell phone | 0.150 |  | 0.100 |  | 0.025 |  | 0.039 |  | 0.111 |  | 0.131 |
| Has internet access | -1.147 | *** | 0.310 |  | -0.156 | *** | 0.046 |  | -0.109 |  | 0.223 |
| Married | -0.658 | * | 0.397 |  | 0.215 |  | 0.167 |  | 3.926 | *** | 0.821 |
| Partner: other wives | 0.265 | * | 0.136 |  | -0.072 |  | 0.057 |  | -0.180 |  | 0.192 |
| Partner: age | 0.208 | *** | 0.077 |  | 0.057 | * | 0.033 |  | -0.137 |  | 0.092 |
| Partner: age is not known | 1.314 | *** | 0.395 |  | 0.253 | * | 0.146 |  | -1.034 | ** | 0.461 |
| Partner: highest education (reference: none) |  |  |  |  |  |  |  |  |  |  |  |
| Primary school | -0.401 | * | 0.230 |  | -0.077 |  | 0.076 |  | 1.112 | *** | 0.342 |
| Middle school | -1.285 | *** | 0.345 |  | -0.045 |  | 0.079 |  | 0.380 |  | 0.348 |
| High school or higher | -0.269 |  | 0.216 |  | -0.107 |  | 0.073 |  | 0.568 | ** | 0.242 |
| Educated, but unsure of grade | -0.203 |  | 0.135 |  | -0.209 | *** | 0.059 |  | 0.426 | *** | 0.164 |
| Partner: works | 0.292 | * | 0.182 |  | -0.052 |  | 0.082 |  | 0.214 |  | 0.233 |
| Number of beds in the home | 0.027 |  | 0.020 |  | 0.029 | *** | 0.007 |  |  |  |  |
| Employ help in the home | -0.216 |  | 0.149 |  | -0.043 |  | 0.043 |  |  |  |  |
| Running water in the home | -0.028 |  | 0.108 |  | 0.051 |  | 0.035 |  |  |  |  |
| Toilet in the home | 0.800 | *** | 0.213 |  | -0.136 | *** | 0.051 |  |  |  |  |
| Constant | -4.416 | *** | 1.582 |  | 4.372 | *** | 0.349 |  | -14.151 | *** | 2.900 |
| *DFRE Variables* |  |  |  |  |  |  |  |  |  |  |  |
| Point 1 (Normalized to Zero) | 0.000 |  | 0.000 |  | 0.000 |  | 0.000 |  |  |  |  |
| Point 2 | -11.079 | *** | 0.437 |  | 4.833 | *** | 0.128 |  | 10.241 | *** | 1.743 |
| Point 3 | 0.418 |  | 0.577 |  | 0.120 |  | 0.085 |  | 10.758 | *** | 1.701 |
| Point 4 | 4.223 | *** | 1.002 |  | -0.059 |  | 0.087 |  | 9.646 | *** | 1.850 |
| District Fixed Effects | Yes | | |  | Yes | | |  | Yes | | |
| Joint Log-Likelihood Function Value | -20679.9 | | | | | | | | | | |
| Observations | 9263 | | |  | 7519 | | |  | 9263 | | |
| *Notes*: An upward testing approach (described in Section 4.4 of the paper) is used to determine the optimal number of mass points. The log-likelihood function value at the maximum for the model with HSEs defined by a 1 KM radius around a PSU centroid is -20677.3. The estimated share of individuals represented by each mass point in the 2 KM model is 48%, 4%, 33%, and 15% respectively. | | | | | | | | | | | |
|  |  |  |  |  |  |  |  |  |  |  |  |
|  |  |  |  |  |  |  |  |  |  |  |  |
| * statistically significant at the 10% level. |  |  |  |  |  |  |  |  |  |  |  |
| ** statistically significant at the 5% level. |  |  |  |  |  |  |  |  |  |  |  |
| *** statistically significant at the 1% level. |  |  |  |  |  |  |  |  |  |  |  |

| **Table F: Results with Additional Kid Wanted** | | | | | | | | | | | |
| --- | --- | --- | --- | --- | --- | --- | --- | --- | --- | --- | --- |
|  | Up to God | | |  | Additional Kid Wanted | | |  | Family Planning Use | | |
|  | Coef. |  | SE |  | Coef. |  | SE |  | Coef. |  | SE |
| *Health Facility and Pharmacy Variables* |  |  |  |  |  |  |  |  |  |  |  |
| Number of public facilities |  |  |  |  |  |  |  |  | 0.019 |  | 0.088 |
| Number of private facilities |  |  |  |  |  |  |  |  | -0.066 |  | 0.117 |
| Number of high volume facilities |  |  |  |  |  |  |  |  | 0.013 |  | 0.114 |
| Number of pharmacies |  |  |  |  |  |  |  |  | -0.009 |  | 0.025 |
| No facilities/pharmacies participate in survey |  |  |  |  |  |  |  |  | -0.730 |  | 0.578 |
| Average number of doctors at facility |  |  |  |  |  |  |  |  | -0.099 |  | 0.224 |
| Average number of nurses at facility |  |  |  |  |  |  |  |  | 0.048 |  | 0.077 |
| Average number of midwives at facility |  |  |  |  |  |  |  |  | -0.058 |  | 0.136 |
| Prop. of pharmacies with mult. pharmacists |  |  |  |  |  |  |  |  | 1.751 | ** | 0.789 |
| Prop. of facilities with a family planning protocol |  |  |  |  |  |  |  |  | 1.007 | ** | 0.490 |
| Prop. of pharmacies requiring FP training |  |  |  |  |  |  |  |  | -0.742 |  | 0.514 |
| Prop. pharmacies allowing staff to advise on FP |  |  |  |  |  |  |  |  | -0.657 |  | 0.477 |
| Average FP methods sold at health facility |  |  |  |  |  |  |  |  | -0.037 |  | 0.100 |
| Average FP methods sold at pharmacy |  |  |  |  |  |  |  |  | 0.137 |  | 0.139 |
| Any facility has an FP social program |  |  |  |  |  |  |  |  | -0.626 |  | 0.429 |
| Any pharmacy has a FP social program |  |  |  |  |  |  |  |  | 0.692 | * | 0.388 |
| Any facility has a health social worker | -0.403 | *** | 0.112 |  | -0.125 |  | 0.078 |  | 0.132 |  | 0.270 |
| Any facility has IEC outreach program | 0.023 |  | 0.090 |  | 0.011 |  | 0.071 |  | -0.334 |  | 0.242 |
| Any facility hosts health talks for the comm. | 0.058 |  | 0.114 |  | -0.006 |  | 0.087 |  | -0.493 |  | 0.380 |
| Prop. giving FP advice during non-FP visit |  |  |  |  |  |  |  |  | 0.120 |  | 0.521 |
| Average number if IEC FP materials at facility |  |  |  |  |  |  |  |  | 0.086 |  | 0.117 |
| Number of health facilities with delivery services | 0.151 | *** | 0.035 |  | 0.024 |  | 0.026 |  |  |  |  |
| *Individual Variables* |  |  |  |  |  |  |  |  |  |  |  |
| Additional kids wanted |  |  |  |  |  |  |  |  | -0.178 | *** | 0.068 |
| Number of kids is left "up to God" |  |  |  |  |  |  |  |  | -0.816 | * | 0.429 |
| Additional kids wanted * married |  |  |  |  |  |  |  |  | 0.191 | ** | 0.078 |
| Number of kids is left "up to God" * married |  |  |  |  |  |  |  |  | 0.029 |  | 0.448 |
| Age (reference: 40+) |  |  |  |  |  |  |  |  |  |  |  |
| 15-19 | -0.485 | *** | 0.125 |  | 2.931 | *** | 0.122 |  | -2.664 | *** | 0.370 |
| 20-24 | -0.541 | *** | 0.116 |  | 2.721 | *** | 0.118 |  | -1.041 | *** | 0.269 |
| 25-29 | -0.298 | *** | 0.109 |  | 2.214 | *** | 0.116 |  | -0.713 | *** | 0.245 |
| 30-34 | -0.631 | *** | 0.109 |  | 1.433 | *** | 0.119 |  | 0.011 |  | 0.206 |
| 35-39 | -0.490 | *** | 0.110 |  | 0.880 | *** | 0.125 |  | 0.193 |  | 0.215 |
| Highest level of education (reference: none) |  |  |  |  |  |  |  |  |  |  |  |
| Primary school | -0.370 | *** | 0.069 |  | -0.172 | *** | 0.062 |  | 0.673 | *** | 0.172 |
| Middle school | -0.664 | *** | 0.116 |  | -0.299 | *** | 0.077 |  | 1.221 | *** | 0.252 |
| High school or higher | -0.657 | *** | 0.158 |  | -0.135 |  | 0.088 |  | 0.688 | ** | 0.270 |
| Ethnicity (reference: other) |  |  |  |  |  |  |  |  |  |  |  |
| Wolof | 0.147 | * | 0.088 |  | 0.136 | ** | 0.061 |  | -0.368 | * | 0.216 |
| Poular | 0.117 |  | 0.097 |  | -0.065 |  | 0.068 |  | -0.298 |  | 0.233 |
| Serer | -0.017 |  | 0.102 |  | 0.124 | * | 0.066 |  | -0.402 | * | 0.223 |
| Socioeconomic status (reference: 1st quintile) |  |  |  |  |  |  |  |  |  |  |  |
| 2nd quintile | 0.079 |  | 0.092 |  | -0.025 |  | 0.076 |  | -0.035 |  | 0.202 |
| 3rd quintile | 0.015 |  | 0.097 |  | 0.154 | ** | 0.077 |  | 0.006 |  | 0.202 |
| 4th quintile | -0.153 |  | 0.109 |  | 0.085 |  | 0.082 |  | -0.099 |  | 0.209 |
| 5th quintile | -0.090 |  | 0.129 |  | 0.196 | ** | 0.091 |  | -0.378 | * | 0.225 |
| Muslim | 0.652 | *** | 0.193 |  | 0.302 | *** | 0.079 |  | -1.054 | *** | 0.287 |
| Worked last week | 0.210 | * | 0.122 |  | -0.056 |  | 0.050 |  | 0.379 | *** | 0.124 |
| Listens to radio | -0.406 | *** | 0.063 |  | 0.121 | ** | 0.053 |  | 0.050 |  | 0.129 |
| Reads the newspaper/magazines | -0.282 | *** | 0.096 |  | 0.009 |  | 0.054 |  | -0.188 |  | 0.181 |
| Has a cell phone | 0.094 |  | 0.066 |  | 0.343 | *** | 0.054 |  | 0.139 |  | 0.135 |
| Has internet access | -0.725 | *** | 0.144 |  | -0.263 | *** | 0.056 |  | 0.018 |  | 0.231 |
| Married | -0.490 | * | 0.256 |  | -0.051 |  | 0.264 |  | 2.695 | *** | 0.652 |
| Partner: other wives | 0.164 | ** | 0.082 |  | -0.122 |  | 0.092 |  | -0.286 | * | 0.171 |
| Partner: age | 0.151 | *** | 0.047 |  | -0.337 | *** | 0.053 |  | -0.144 |  | 0.094 |
| Partner: age is not known | 0.939 | *** | 0.220 |  | -1.315 | *** | 0.218 |  | -1.030 | ** | 0.461 |
| Partner: highest education (reference: none) |  |  |  |  |  |  |  |  |  |  |  |
| Primary school | -0.292 | ** | 0.133 |  | 0.211 | * | 0.116 |  | 0.998 | *** | 0.308 |
| Middle school | -0.904 | *** | 0.182 |  | 0.233 | * | 0.126 |  | 0.430 |  | 0.373 |
| High school or higher | -0.171 |  | 0.131 |  | 0.607 | *** | 0.106 |  | 0.795 | *** | 0.317 |
| Educated, but unsure of grade | -0.149 | * | 0.086 |  | 0.254 | *** | 0.093 |  | 0.488 | *** | 0.177 |
| Partner: works | 0.210 | * | 0.122 |  | 0.035 |  | 0.140 |  | 0.041 |  | 0.247 |
| Number of beds in the home | 0.020 |  | 0.013 |  | 0.018 | * | 0.010 |  |  |  |  |
| Employ help in the home | -0.141 |  | 0.092 |  | 0.044 |  | 0.058 |  |  |  |  |
| Running water in the home | -0.016 |  | 0.067 |  | 0.144 | *** | 0.047 |  |  |  |  |
| Toilet in the home | 0.378 | *** | 0.103 |  | -0.138 | ** | 0.070 |  |  |  |  |
| Constant | -1.719 | *** | 0.336 |  | 0.556 | *** | 0.189 |  | -0.689 |  | 0.612 |
| *DFRE Variables* |  |  |  |  |  |  |  |  |  |  |  |
| Point 1 (Normalized to Zero) | 0.000 |  | 0.000 |  | 0.000 |  | 0.000 |  | 0.000 |  | 0.000 |
| Point 2 | -11.241 | *** | 0.319 |  | 5.967 | *** | 0.190 |  | -3.096 | *** | 0.753 |
| Point 3 | 0.082 |  | 0.318 |  | 0.736 | *** | 0.102 |  | -13.647 | *** | 1.333 |
| District Fixed Effects | Yes | | |  | Yes | | |  | Yes | | |
| Observations | 9263 | | |  | 7519 | | |  | 9263 | | |

*Notes:* This table contains parameter estimates from our full model, with unobserved heterogeneity, where the “ideal number of kids” outcome variable has been replaced by “additional kids wanted.” Adding a fourth unobserved mass point did not generate a significant improvement in the log-likelihood function value.

* statistically significant at the 10% level.

** statistically significant at the 5% level.

*** statistically significant at the 1% level.

1. Parameter estimates for the three models without district-level dummies can be seen in Table A below. Parameter estimates for the contraceptive use, ideal number of children, and up to God equations with district-level dummies can be seen in the left-most columns of Tables 5, 6, and 7, respectively. [↑](#footnote-ref-1)
2. Note that the parametric bootstrap procedure that we employ in simulation requires sampling parameters from a multivariate normal distribution centered at the point estimates of the coefficients, which utilizes the estimated covariance matrix. Since the focus is on whether or not simulated effects are significantly different with and without the corrections for unobserved heterogeneity, we use the joint covariance matrix for parameters estimated with and without unobserved heterogeneity in order to explicitly control for the covariance between these two sets of results (see Mroz, 1987). [↑](#footnote-ref-2)
